# Comprehensive mapping of genetic variation at Epromoters reveals pleiotropic association with multiple disease traits

**DOI:** 10.1093/nar/gkae1270

**Published:** 2024-12-27

**Authors:** Jing Wan, Antoinette van Ouwerkerk, Jean-Christophe Mouren, Carla Heredia, Lydie Pradel, Benoit Ballester, Jean-Christophe Andrau, Salvatore Spicuglia

**Affiliations:** Aix-Marseille University, INSERM, TAGC, UMR 1090 Marseille, France; Equipe Labellisée LIGUE, 2023 Marseille, France; Aix-Marseille University, INSERM, TAGC, UMR 1090 Marseille, France; Equipe Labellisée LIGUE, 2023 Marseille, France; Aix-Marseille University, INSERM, TAGC, UMR 1090 Marseille, France; Institut de Génétique Moléculaire de Montpellier, University of Montpellier, CNRS, UMR 5535, Montpellier, France; Aix-Marseille University, INSERM, TAGC, UMR 1090 Marseille, France; Equipe Labellisée LIGUE, 2023 Marseille, France; Aix-Marseille University, INSERM, TAGC, UMR 1090 Marseille, France; Institut de Génétique Moléculaire de Montpellier, University of Montpellier, CNRS, UMR 5535, Montpellier, France; Aix-Marseille University, INSERM, TAGC, UMR 1090 Marseille, France; Equipe Labellisée LIGUE, 2023 Marseille, France

## Abstract

There is growing evidence that a wide range of human diseases and physiological traits are influenced by genetic variation of *cis*-regulatory elements. We and others have shown that a subset of promoter elements, termed Epromoters, also function as enhancer regulators of distal genes. This opens a paradigm in the study of regulatory variants, as single nucleotide polymorphisms (SNPs) within Epromoters might influence the expression of several (distal) genes at the same time, which could disentangle the identification of disease-associated genes. Here, we built a comprehensive resource of human Epromoters using newly generated and publicly available high-throughput reporter assays. We showed that Epromoters display intrinsic and epigenetic features that distinguish them from typical promoters. By integrating Genome-Wide Association Studies (GWAS), expression Quantitative Trait Loci (eQTLs) and 3D chromatin interactions, we found that regulatory variants at Epromoters are concurrently associated with more disease and physiological traits, as compared with typical promoters. To dissect the regulatory impact of Epromoter variants, we evaluated their impact on regulatory activity by analyzing allelic-specific high-throughput reporter assays and provided reliable examples of pleiotropic Epromoters. In summary, our study represents a comprehensive resource of regulatory variants supporting the pleiotropic role of Epromoters.

## Graphical Abstract

**Graphical Abstract:**
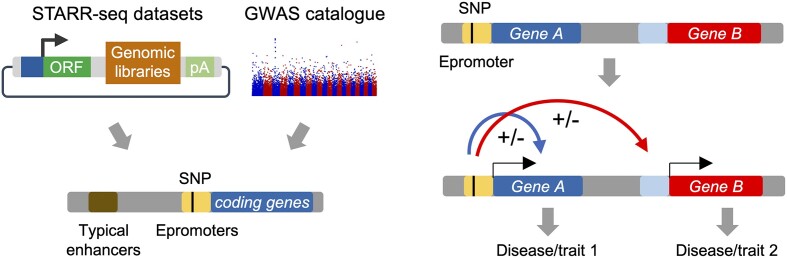

## Introduction

In higher eukaryotes, gene transcription is regulated through the involvement of regulatory elements that are located near the transcription start site (TSS), called promoters, and those that are located far from TSS, called enhancers. This classical definition implies that enhancers activate gene expression at a distance while promoters induce local gene expression. However, several lines of evidence have now established that some coding-gene promoters, termed Epromoters, also work as *bona fide* enhancers in different cellular contexts from drosophila to humans (1–10). These promoter elements can regulate distal promoters when assessed in episomal reporter systems or high-throughput reporter assays (1,2,4,6,9,11). More importantly, their deletion or epigenetic silencing in their natural context results in the loss of expression of distal genes (5–9). Subsequent studies have shown that Epromoters work as a hub for recruiting essential transcription factors (TFs) required for gene activation in different inflammatory and stress conditions and establishing connections with other distal response genes within the same clusters to ensure a rapid coordinated expression response (6,9). Although typical enhancers and promoters are generally distinguished by their relative location to the TSS of genes they regulate, their shared architectural properties have suggested a unifying model of gene regulation by *cis*-regulatory elements (10,12–16). Previous studies have suggested that Epromoters share functional and architectural properties with both types of *cis*-regulatory elements (10,12,13). Epromoters have been associated with a higher density of TFs and co-activators binding, higher levels of unstable bidirectional transcripts and more frequent promoter–promoter (P–P) interactions (6,10,17). However, a more systematic comparison between typical promoters and Epromoters should provide a better understanding of the intrinsic features driving the dual enhancer and promoter function of Epromoters.

There is growing evidence that a wide range of human diseases is influenced by the dysfunction of *cis-*regulatory elements caused by genetic, structural or epigenetic mechanisms (18). These processes frequently underpin the susceptibility to common diseases but can be also directly involved in cancer or Mendelian diseases. The advent of genome-wide association studies (GWASs) in the past decade has been a great endeavor in genomic research toward identifying genetic variants associated with candidate genes for common diseases. The majority of these genetic variants are found in non-coding regions and, therefore, are likely to be involved in regulatory mechanisms controlling gene expression (19–21). However, a major challenge in interpreting the impact of genetic mutation or variation in disease is to identify the targets that are impacted by the genomic alteration, which might not necessarily be the closest genes and might have confounding features (22). Despite this, most studies select the closest gene to the associated GWAS variant to establish possible causal mechanisms, namely when the variant lies in the vicinity of a TSS or within an intronic region. However, GWAS variants might regulate the expression of distal disease-causing genes, in particular when lying within Epromoters.

The discovery of Epromoters thus opens a new paradigm in the study of regulatory variants. Previous studies have indicated that Epromoters are more frequently associated with distal expression Quantitative Trait loci (eQTLs) that can potentially influence the expression of distal genes (6,17,23–25). Therefore, genetic variation mutation in an Epromoter could affect the expression of several genes or change the relative ratio of promoter versus enhancer activity. This could result in a variety of potential changes in the relative expression of neighboring genes. In addition, it is plausible that the same *cis*-regulatory element displays preferential promoter activity in some tissues while displaying increased enhancer activity in other tissues, depending on the expressed combination of TFs and the epigenetic context (6,26,27). Given the potential regulation of proximal and distal genes by Epromoters, we hypothesized that genetic variation or mutation at Epromoters might therefore impact several physiological and pathological traits simultaneously.

To better assess the functional properties of Epromoters and the impact of genetic variation on physiological traits and diseases, we generated a comprehensive resource of human Epromoters by combining published and newly generated STARR-seq data from different cell lines and conditions. Epromoters displayed intrinsic genomics and epigenomics features that distinguish them from typical promoters. Furthermore, we found that Epromoters have a higher probability of being associated with multiple different GWAS traits, suggesting they are more pleiotropic. Strikingly, Epromoter pleiotropy was found to be associated with distal gene regulation and functional regulatory variants. Our finding supports the hypothesis of an important and pleiotropic role of Epromoter variation on the ontogeny of different diseases and physiological traits.

## Materials and methods

### Cell culture

K562, CCRF-CEM and RPMI cells were maintained in RMPI 1640 medium GlutaMAX (Gibco, 61870010) supplemented with 10% FBS (Gibco, Fetal Bovine Serum A5256701) (inactivated at 55°C for 1 hour) between 0.3 × 10^6^ and 1 × 10^6^ cells per mL, incubated at 37°C with 5% CO_2_. GM12878 cells were maintained in the same conditions but with 15% instead of 10% FBS. Cells were tested for mycoplasma infection once a month and tested negative. A549 cells were maintained in DMEM/F12 GlutaMAX (Gibco, 10565018) supplemented with 10% deactivated FBS, incubated at 37°C with 5% CO_2_. When 90% confluent, the medium was aspirated and cells rinsed with phosphate-buffered saline (PBS), followed by trypsinization (Trypsin-EDTA (0.05%), phenol red, Gibco, 25300–062) at 37°C for 5 min. 5x the volume of medium is added to detach the cells from the dish, the cells are centrifuged, resuspended in the medium and split at the appropriate density into a new dish. Cells were tested for mycoplasma infection once a month and tested negative.

### CapSTARR-seq

The human promoter CapSTARR-seq library used in this study has been generated previously (6,9). The STARR-seq protocol was performed in CCRF-CEM (without stimulation and with interferon alpha (IFNa) stimulation), RPMI and GM12878 cell lines. Around 100 million cells were transfected with 1.25 mg of CapSTARR-seq promoter library using the Neon transfection system (Thermo Fisher Scientific) using the following settings: voltage V 1300, pulse width 20 and pulse number 3. After 24 h of incubation, either the STARR-seq protocol was performed as published before (6), or (for CCRF-CEM cells) IFNa was used to induce interferon response (100 ng/mL, Sigma Aldrich, SRP4594) for 6 h followed by the STARR-seq protocol (9). cDNA and input libraries were sequenced on an Illumina NextSeq500, and mapping and analysis were performed as published (9).

### STARR-seq and CapStarr-seq data processing

Human enhancers were retrieved from 19 whole-genome STARR-seq, 2 ChIP-STARR-seq and 7 CapStarr-seq datasets (Supplemental Table S1). Seventeen whole genome STARR-seq datasets (A549, MCF-7, HCT116, SH-SY5Y, HepG2 and K562 with different stimulation) were obtained from ENCODE and were already processed by STARRPeaker (28) or MACS2 (29) for peak calling defining the active enhancer regions. The peak files in bed format were directly downloaded from ENCODE (ENCODE accessions in Supplemental Table S1). To recover high-quality peaks, we took common peaks from different replicates for each dataset and averaged the enhancer activity values. Common peaks were ranked by the average values and peaks with the values higher than the inflection point (inflection R package) were taken as enhancers in this study. Two whole genome STARR-seq datasets in Hela were collected from supplementary data (GSE100432) of Muerdter et al. (11). Two ChIP-STARR-seq datasets in hESC were collected from supplementary data of Barakat et al. (30) (GSE99631). The two hESC datasets were filtered by at least one of the active regions of NANOG, OCT4, H3K27ac and H3K4me1, with the enhancer activity score RPP (reads per plasmid) over 256 according to the original analysis described in (30). Three Capstarr-seq datasets in Hela and K562 were collected as Epromoters from supplementary data of Dao et al. (6) and Santiago et al. (9). Four Capstarr-seq datasets in GM12878, CCRF-CEM (with and without IFNa stimulation) and RPMI were generated in this study (GEO accession numbers are provided in Supplemental Table S1) and processed as previously described (6,9). Briefly, fastq files were trimmed using sickle with -q 20 option and mapped to the hg19 reference genome using Bowtie2 with default parameters. Sam files were converted using SamTools and bed files were generated with bedtools ‘BamToBed’ command. Fragment reads were extended to 314 nt, corresponding to the average size of the captured fragments. Coverage of captured regions was computed using bedtools ‘coverage’ command for both transfected and non-transfected libraries. The coverage was normalized by Fragments per kilobase per million reads mapped (FPKM). Promoter regions with an FPKM < 1 in the input library were removed. The ratio of the Capstarr-seq coverage over the input (fold-change) was computed for each sample. Promoter regions with enhancer activity were defined using the inflection point of the ranked fold-change as a threshold. Finally, all the enhancer regions from the 28 datasets were converted to hg38 coordinates and merged into a single non-redundant list in bed format. This resulted in 58388 non-redundant enhancers in 11 cell lines (Supplemental Table S2 and Supplemental Table S3).

### Epromoter identification

To identify Epromoters in the human genome, first, we defined the promoter region according to the hg38 genome annotation file from Ensembl (release-103, http://ftp.ensembl.org/pub/release-103/gtf/homo_sapiens/). The promoters were defined as 500-bp region upstream of the TSS of each protein-coding transcript. The promoter regions were overlapped with no-redundant enhancers by bedtools intersect (v2.28.0) (31), with at least 50% overlap (bedtools intersect -wa -wb -f 0.5 -F 0.5 -e). The enhancer-overlapping promoters were defined as Epromoters. The Epromoter regions were merged if they overlapped by at least 1 nt. Finally, 5743 non-redundant Epromoters were defined (Supplemental Table S2 and Supplemental Table S3).

### Gene expression and tissue specificity calculation

Gene expression data was downloaded from the supplementary data of Uhlén et al. (32) (Table EV1). The study provided a gene expression matrix of 18684 genes across 30 human tissues from GTEx. The tissue specificity was calculated according to Yanai et al. (33), using the following formula:


\begin{eqnarray*}Tissue\ specificity\ index = \frac{{\mathop \sum \nolimits_{i = 1}^N \ \left( {1 - {{x}_i}} \right)}}{{N - 1}},\end{eqnarray*}

where N is the number of tissues and x_i_ is the expression profile component normalized by the maximal component value. The tissue specificity index varies from 0 to 1, where 0 means broad expression and 1 means high specificity.

### Control promoter set

We generated a control promoter set associated with genes displaying the most similar expression patterns as the Epromoter-associated genes. First, all coding genes were clustered according to the gene expression across 30 tissues using the expression matrix from (32) (Hierarchy cluster was performed with the ‘Euclidean’ method in R4.3.2). For each Epromoter-associated gene, the gene that is nearest to the Epromoter gene in the cluster results was assigned as a control gene. The control promoter regions were defined as described for Epromoters.

### P–P interactions analysis

The P–P interaction data were collected from two promoter capture-Hi-C studies (24,34) and the ABC model predictions (35). We downloaded the processed high-confidence interactions (CHICAGO score ≥ 5) from Supplemental Data S1 of (34), which was generated by promoter capture-Hi-C from 17 blood cell types. The data from (24) were downloaded from their Supplementary Table 4, which includes processed significant P–P capture-Hi-C interactions from 26 human tissues. Nasser et al. provided a comprehensive element-gene connections resource across 131 human cell types and tissues by the ABC model, which is a high-performance prediction model based on measurements of chromatin accessibility, H3K27ac and Hi-C data (35). The ABC predictions in 131 cell types and tissues were downloaded from (https://www.engreitzlab.org/resources). After converting to hg38, all interactions from the three datasets were overlapped with total promoters (5′ upstream 500bp of TSS, Ensembl) in both anchors as the total P–P interactions. The target genes of Epromoters and control promoters were identified by overlapping their coordinates with the total P–P interactions, which include the target genes associated with each promoter. The circular visualization and P–P interactions in the Epromoter instances was performed by R package circlize (36).

### CRISPRi screen analysis

CRISPRi-based inactivation (CRISPRi) screen data were collected from Replogle et al. (37) and Gasperini et al. (38). Replogle et al. generated genome-scale CRISPRi screen data in K562 by Perturb-seq. We downloaded the processed Perturb-seq file of K562 genome-scale sample in h5ad format (https://gwps.wi.mit.edu/, gemgroup Z-normalized pseudo-bulk expression data). The processed Perturb-seq file was processed by Seurat (V5) (39) into a normalized expression matrix of all gRNAs and effect genes. We first identified a set of 5054 promoters that had been efficiently inactivated (i.e. the associated gene is among the top 2 of repressed genes). The top 30 repressed genes were taken as regulated genes of these promoters. We then identified the promoters for which CRISPRi resulted in the repression of *cis*-distal genes (<1 Mb). This CRISPRi result was intersected with Epromoters and control promoters to identify their *cis*-regulated genes. Gasperini et al. performed CRISPRi perturbations in K562, which include target sites on TSS as positive controls. We downloaded the CRISPRi screen results from the pilot and scale experiments (https://www.ncbi.nlm.nih.gov/geo/query/acc.cgi?acc=GSE120861). The results include the target sites' position and expression-affected genes. The target sites of positive controls were extracted to overlap with Epromoters. The expression-affected genes that were not on the target sites were taken as distal effect genes of Epromoters. The same analysis was performed for control promoters.

### Chromatin state enrichments

The chromatin state annotation data was downloaded from full-stack ChromHMM model trained with 1032 datasets from 127 reference epigenomes (https://public.hoffman2.idre.ucla.edu/ernst/2K9RS/full_stack/full_stack_annotation_public_release/hg38/) (40). The full-stack annotations in bed format were overlapped with Erpromoters and control promoters by command ‘ChromHMM.jar OverlapEnrichment’ (41). In this command, the fold enrichment was calculated as the ratio of the proportion of overlapping bases in the state to the proportion of bases in the external annotation, relative to the total genome size.

### Sequence conservation analysis

The sequence conservation was evaluated by three different methods, including phyloP (42), PhastCons (43) and Combined Annotation Dependent Depletion scores (CADD) (44). For phyloP, the sequence conservation data was downloaded from the Zoonomia Placental Mammals track (including 241 vertebrate species) (45) in the UCSC genome browser (https://hgdownload.soe.ucsc.edu/goldenPath/hg38/cactus241way/cactus241way.phyloP.bw). The conservation scores included in the bigwig file were computed by phyloP (42) from the PHAST package (43) at each single nucleotide level. In this conservation score, each base with positive scores was predicted as conserved, and negative scores were predicted as fast-evolving. The bigwig file of conservation scores was converted into wig format by ‘bigWigToWig’ and then into bed file by ‘wig2bed’. The conservation score of each Epromoter was calculated by the sum of all the bases. For PhastCons, the sequence conservation data were downloaded from the Multiz 470-way track (470 mammals) in the UCSC genome browser (https://hgdownload.soe.ucsc.edu/goldenPath/hg38/phastCons470way/hg38.phastCons470way.bw). Similarly, the conservation scores in bigwig were overlapped with Epromoters and control promoters. For CADD, the mutation effect data were downloaded from CADD website in version 1.7 (https://kircherlab.bihealth.org/download/CADD/bigWig/CADD_GRCh38-v1.7.bw). The mutation effect scores in bigwig were overlapped with Epromoters and control promoters.

### CpG island and G4 analysis

The CpG islands (CGIs) annotations have been recovered from UCSC (https://genome.ucsc.edu/cgi-bin/hgTables?hgta_doMainPage=1&hgta_group=regulation&hgta_track=cpgIslandExt&hgta_table=cpgIslandExt&hgsid=1956573466_K6emxl9N7oynnWsuT8Zjnyk9XX6n) in hg38 genome version. This dataset contains CGIs ‘masked’ that do not contain repetitive elements. CGIs in Epromoter or control promoters were identified by using the Bedtools (2.31.0). The coverage of G-quadruplexes (G4) in Epromoters or control promoters was calculated as the percent of base pairs covered by predicted G4 annotations. These annotations and G4 Hunter scores are obtained from the G4Hunter algorithm described in Bedrat et al. (46) by using the threshold score 1. The statistical significance was calculated by R with the Kolmogorov–Smirnov test.

### TF binding analysis

TF binding sites (TFBS) data were collected from the JASPAR (2022) database (47), which was downloaded in bigbed format (http://hgdownload.soe.ucsc.edu/gbdb/hg38/jaspar/JASPAR2022.bb) from the UCSC genome track with a score of *P*-value for each binding site. All the TFBSs were filtered by a score higher than 400 (*P*-value ≤ 10e-4). The filtered TFBSs were overlapped with Epromoters by bedtools intersect. Each TFBS was associated with a corresponding TF family according to the supplemental data from Castro-Mondragon et al. (47). The TFBS family density was calculated by the binding sites of TF families at each Epromoter. The TFBS family diversity was calculated by the number of TF families at each Epromoter. The same analysis was performed for control promoters. TF binding data were collected from ReMap (2022) (48). We used the ReMap datasets that include 68.2 million non-redundant ChIP-seq peaks from 1210 TFs in humans (https://zenodo.org/records/10527088). The non-redundant ChIP-seq peaks were overlapped with Epromoters to quantify the number of peaks per Epromoter. 737 cell lines and tissues associated with the ChIP-seq peaks were classified into 18 biotypes to describe TF diversity (48). The same analysis was also performed for control promoters. The odds ratio and *P*-value were calculated for each TF between Epromoters and control promoters by the number of ChIP-seq peaks, as the description of TFs binding enrichment at Epromoters. The uniform manifold approximation and projection (UMAP) analysis was performed by the R package umap, which is based on a matrix of each TF binding state (ReMap) at each Epromoter or control promoter (Value 1 is defined as binding, and value 0 is defined as no-binding). Then each Epromoter or control promoter was quantified by the TF binding peak density.

### CAGE data analysis

The CAGE data were collected from FANTOM5 (49–51). The CAGE peaks were downloaded in bed format with hg38 (https://fantom.gsc.riken.jp/5/datafiles/reprocessed/hg38_v7/extra/CAGE_peaks/), which was identified by DPI (decomposition-based peak identification, Forrest et al 2014) across all the tissues in FANTOM5. The CAGE signal data were downloaded from the UCSC track in bigwig format (https://hgdownload.soe.ucsc.edu/gbdb/hg38/fantom5/ctssTotalCounts.fwd.bw, https://hgdownload.soe.ucsc.edu/gbdb/hg38/fantom5/ctssTotalCounts.rev.bw), which include the total reads count by strand across all tissues from FANTOM5. In the CAGE signal analysis, we defined forward signal as direction (strand) consistent between the CAGE signal and genes and reverse signal as inconsistent. Epromoters were extended to 500-bp upstream and downstream of TSS to cover the forward and reverse signals around TSS. The stranded sense and antisense CAGE peaks were overlapped with the extended regions of Epromoters to address the directionality. The CAGE signal in bigwig was overlapped with the extended regions of Epromoters by strand separately to quantify the transcription initiation. The same analysis was performed for control promoters.

### RNAPII data analysis

RNAPII data were collected from de Langen et al. (52) (https://zenodo.org/records/8091826), which include RNAPII consensus peaks identified from 900 RNAPII ChIP-seq experiments in normal tissues and cancer samples. The RNAPII consensus peaks were overlapped with Epromoters to quantify the RNAPII enrichment from different tissues and samples. The same analysis was performed for control promoters.

### Distal enhancer set

The distal enhancers were defined by the intersection between Candidate Enhancers from ENCODE (https://downloads.wenglab.org/cCREs/GRCh38-ELS.bed) (53) and STARR-seq dataset in this study. First, the candidate distal enhancers were taken from STARR-seq dataset by excluding Epromoters. Then, the ENCODE enhancers were overlapped with the candidate distal enhancers by bedtools with at least 50% overlapping rate. The center 500 bp of intersected enhancers from ENCODE were defined as the distal enhancers. In total, 350 07 distal enhancers were retrieved.

### Common SNPs and rare SNPs collection

The total single nucleotide polymorphisms (SNPs) (660 146 174 SNPs) were downloaded from SNPdb in VCF format in hg38 (https://ftp.ncbi.nlm.nih.gov/snp/organisms/human_9606/VCF/00-All.vcf.gz). The common (37), 302 978) and rare (45), 894 070) SNPs were filtered by minor allele frequency (MAF) of more or less than 1% according to 1000 genomes allele frequency, respectively. The common and rare SNPs were overlapped with Epromoters and control promoters by bedtools intersect.

### GWAS analysis

Around 186 120 GWAS variants associated with 4138 GWAS traits were collected from the NHGRI-EBI GWAS Catalog (v1.0.2) (https://www.ebi.ac.uk/gwas/api/search/downloads/alternative) (54). SNPs without rsID and genomic coordinates were removed. The human common SNPs were downloaded from 1000 Genomes Project (v5a) in vcf format (http://ftp.ensembl.org/pub/data_files/homo_sapiens/GRCh38/variation_genotype/) (55), which were filtered by Plink (v1.9) (56) from 5 super populations (European, African, American, East Asian and South Asian) using the following parameters: the proportion of missing genotypes 5%, MAE 1%, Hardy-Weinberg equilibrium 1e-6. The lead SNPs from GWAS Catalog were linked with common SNPs from 1000 Genomes Project by Plink with parameters of –ld-window-kb 1000 –ld-window-r2 0.8, allowing to retrieve SNPs within 1 Mb in high linkage disequilibrium (r2 > 0.8) of each lead SNP. Then these linkage disequilibrium SNPs associating with GWAS (GWAS-SNPs) were overlapped with Epromoters and control promoters. Each GWAS study was assigned a GWAS trait with unique EFO (Experimental Factor Ontology) ID (https://www.ebi.ac.uk/ols4/ontologies/efo)(57). Each GWAS trait was mapped into a parent trait, including 17 categories according to the EFO database. The number of GWAS traits associated with each promoter was counted by the total non-redundant GWAS traits of different SNPs at the same promoter. The GWAS trait enrichment was calculated by the ratio of SNPs associating each GWAS trait between Epromoters or control promoters versus whole genome (hypergeometric test). The GWAS trait enrichment was also compared between Epromoters and control promoters (Chi-Squared test). The GWAS category enrichment was calculated in the same way as GWAS trait enrichment. The GWAS results were additionally filtered by excluding ‘Biological process’, ‘Body measurement’, ‘Other measurement’, ‘Other trait’ from 17 categories, to take the remaining 14 categories as disease-associated categories. Partitioned heritability was calculated by LD score regression (LDSC) (58). We calculated the partitioned heritability of 176 GWAS summary statistics (https://console.cloud.google.com/storage/browser/broad-alkesgroup-public-requester-pays/LDSCORE?pageState) in Epromoters and control promoters, also including the partitioned regions Enhancer_Andersson, Promoter_UCSC, Coding_UCSC annotated by the baseline model of LDSC. For each GWAS study, the partitioned heritability described how much genetic contribution by different partitioned regions.

### eQTL data analysis

The eQTL data were downloaded from the fine-mapped credible sets in eQTL Catalogue (59) (https://www.ebi.ac.uk/eqtl/Data_access/), which used the fine mapping model SuSiE (60). The eQTL data include 9137260 eQTLs identified from 96 tissues or cell types. These eQTLs overlapped with Epromoters and control promoters. The eQTLs associated with different target genes from different tissues were merged into a non-redundant eQTL list. Then, the eQTLs were associated with the GWAS traits by the coordinates overlapping between eQTLs and GWAS-SNPs. We classified the merged eQTL list into three categories by the distance between eQTLs and the TSS of target genes, including proximal eQTLs, distal eQTLs, and proximal and distal eQTLs. The proximal eQTLs were defined as located less than 2 kb from the TSS of all target genes. The distal eQTLs were defined as located more than 2 kb from the TSS of all target genes. The proximal and distal eQTLs were defined as including both proximal and distal target genes. The eQTL heatmap in the Epromoter instances was performed according to the z-score of effect genes associating with each eQTL in different tissues from eQTL Catalogue.

### MPRA resource collection

Massively parallel reporter assays (MPRA) data were collected from 17 published studies (61–77), including 24 MPRA datasets from 14 human cell lines (Supplemental Table S7). We collected the SNPs tested in MPRA from the supplemental data of each study. The assessed SNPs were filtered by the allelic impact thresholds described in the original studies. This resulted in 37 829 SNPs with significant allelic impact overlapped.

### SNP-SELEX collection

The SNP-SELEX data was collected from (78), which systematically assessed the binding of 270 human TFs to 95886 noncoding variants in the human genome using an ultra-high-throughput multiplex protein–DNA binding assay. In the original results, 11 079 SNPs exhibited significantly differential binding to at least one TF. We collected these SNPs with TF binding effect to overlap with Epromoters and control promoters.

### TF binding effect analysis

The TF binding effect analysis of SNPs was analyzed by ANANASTRA (79) (https://ananastra.autosome.org/), which is based on allele-specific binding data from ChIP-Seq. The SNPs at Epromoters and control promoters were loaded into ANANASTRA for analysis by rsID. The parameter of ANANASTRA was the default on the website. Additionally, we used SNP2TFBS (80) (https://epd.expasy.org/snp2tfbs/) and FABIAN-variant (81) (https://www.genecascade.org/fabian/), which based on position weight matrix (PWM) to predict the TF binding effect. The parameters of SNP2TFBS were used default in the websites. The results of FABIAN-variant were filtered by the absolute value of the prediction score over 0.5 for each motif.

### Luciferase reporter assays

Luciferase vectors were generated by GeneCust, inserting the 726 bp *OAS3* promoter region (hg38 chr12:112938128–112938853) with the five minor alleles or the five major alleles into pGL4.12 luc2cp using KpnI-XhoI sites to assess promoter activity, and into pGL4 sv40 luc2cp (9) using BamHI-SalI sites to assess enhancer activity. Sequences of the plasmids are available in Supplemental Table S8. For K562, 3 × 10^6^ cells were spun down per plasmid transfection (3 replicates), and cells were washed with PBS and resuspended in 30 μl Buffer R of the Neon transfection kit (Thermo Fisher Scientific). Around 1 ug of the plasmid to be tested, and 200 ng of Renilla was transfected per 1 × 10^6^ cells in triplicate with the 10 ul NEON tip using the following settings; Voltage: 1450, ms: 10, pulses: 3. 1 × 10^6^ transfected cells were transferred to 2 mL prewarmed medium in a 12-well plate. After 18 h, 1 mL of each transfection was transferred to a new 12-well plate, allowing 1 mL of cells as non-stimulated control, and 1 mL to be treated with human recombinant IFNa protein (100 ng/mL) (Abcam ab9642) for 6 h. For A549, 0.25 × 10^6^ of cells were seeded in a 12-well plate 24 h before transfection. At 90% confluence, the following day, 1 ug of each of the 4 plasmids (promoter and enhancer tests of major and minor *OAS3* haplotype) and 200 ng Renilla were transfected in six wells using the Lipofectamine 3000 (Thermo Fisher Scientific, L3000008) protocol. Twenty-four hours after transfection, three wells were treated with IFNa (100 ng/mL) for 6 h, leaving three wells per plasmid untreated as non-stimulated controls. After 6 h of IFNa stimulation, the cells were washed with 1X PBS, and resuspended in 350 ul lysis buffer of the Dual-Glo Luciferase Assay kit (Promega E2920). After 15 min of incubation in lysis buffer, cells were spun down and 20 ul supernatant was transferred to the luminescence plate reader. Luciferase signal was measured by the addition of 100 ul Luciferin, followed by Renilla signal measurement by the addition of 100 μl STOP&GLO (Promega E2920). The transfection of 3 replicates was repeated once in a separate experiment (to give a total of six samples per construct). For data analysis of the luciferase assays, luciferase values were normalized to the Renilla luciferase activity to control for between-well transfection efficiency. For each construct, readings from different days were merged by normalizing the activity of reporters to the minor allele only vector (reference allele).

### Allelic-specific CapSTARR-seq analysis

Using the BAM files of the CapSTARR-seq data from K562 and CCRF-CEM cell lines with and without IFNa stimulation, the number of reads containing the minor (T) or major (C) allele of the rs1156361 SNP was quantified using the IGV web tool (82). Average read numbers from two replicates were calculated, and the reads were normalized to the no-stimulation condition for each allele.

## Results

### A comprehensive resource of human epromoters

**Figure 1.**
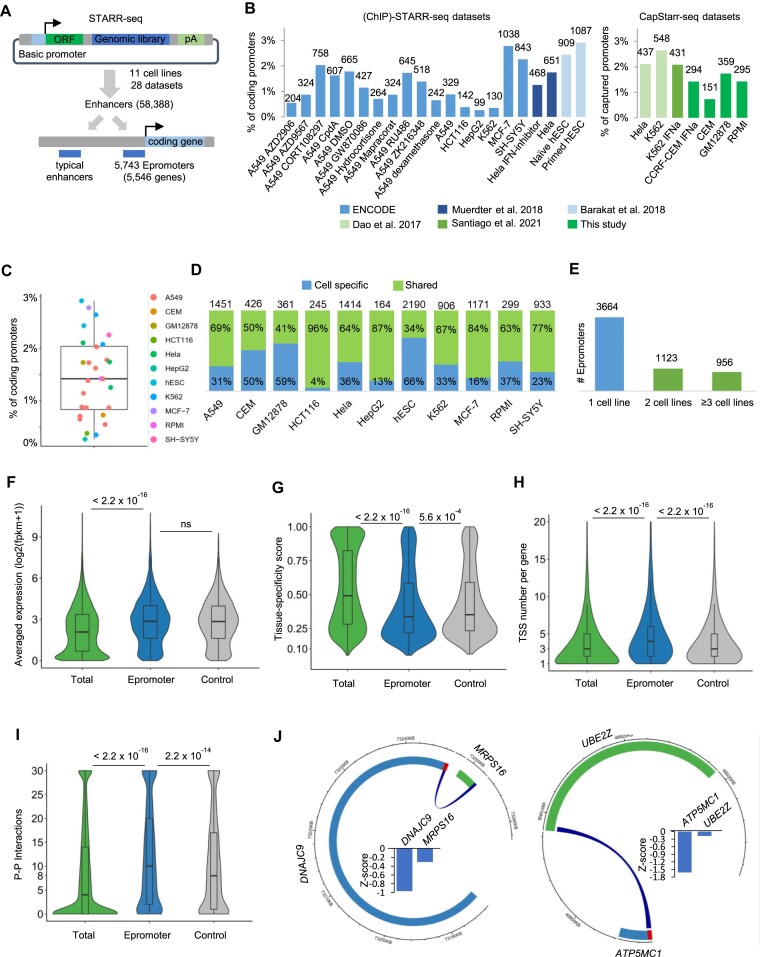
A comprehensive dataset of human Epromoters. (**A**) A schematic diagram illustrating the strategy to identify Epromoters from the (Cap)STARR-seq data. (**B**) The percentage and number of promoters identified as Epromoters identified in each (Cap)STARR-seq dataset are indicated. The legend at the bottom describes the source of the datasets. (**C**) The boxplot shows the percent distribution of promoters identified as Epromoters in each dataset. Each dot represents one dataset as indicated in the legend. (**D**) The bar plots show the percentage of Epromoters found in only one cell line or shared between two or more cell lines. The number of Epromoters in each cell line is shown at the top of each bar. (**E**) The bars show the number of Epromoters found in the indicated number of cell lines. (**F–I**) Violin plots displaying the average gene expression level (**F**), tissue specificity score (**G**), the number of TSS per gene (**H**) and the P–P interactions (**I**) of all protein-coding (Total, 18351), Epromoter-associated (5331), and control genes (5331). The expression for each gene in (**F**) was calculated by the average level across 30 human tissues from GTEx. *P*-values, represented by the numbers in the graphs, were calculated by a Wilcoxon test (ns: not significant). (**J**) Two examples of consistent P–P interactions and CRISPRi-mediated regulation of distal genes by Epromoters. The plots show the circular visualization of Epromoters and interacting genes based on their genomic locations. Epromoter-associated genes are in blue, while the red bar represent the Epromoter. Genes in the outer circle are in the positive strand. Genes in the inner circle are in the negative strand. The blue curves are P–P interactions. The inset plots display the Z score values of the Perturb-seq experiments (37).

To recover active enhancer regions in different cell types we recovered whole genome STARR-seq, ChIP-STARR-seq and CapSTARR-seq experiments from 28 datasets comprising 11 human cell lines and stimulatory conditions, including IFNa and multiple drug treatments (Supplemental Table S1). We retrieved a total of 58 388 non-redundant STARR-seq enhancers. We defined Epromoters as genomic regions of 500 bp upstream of the TSS of any coding gene that overlapped an active enhancer as defined by the STARR-seq assays (Figure 1A). The percentage of active enhancers that were defined as Epromoters ranged from 2.3% to 35.0% depending on the STARR-seq dataset (Supplemental Figure S1A). This resulted in a non-redundant set of 5743 Epromoters, associated with 5546 genes, and representing 15.4% of total coding-gene promoters (Supplemental Table S2). The percentage of Epromoters in each cell type/condition ranged from 0.3% to 2.9% (Figure 1B; Supplemental Table S3), with, on average, 1.5% of total coding gene promoters per experimental dataset (Figure 1C). The differences in the proportion of Epromoters between the datasets are likely explained by the different types of STARR-seq approaches (whole-genome, ChIP-based or array-capture), as well as, the different thresholds applied by the independent studies. For the majority of cell lines, more than 50% of Epromoters were also an Epromoter in at least one other cell line (Figure 1D). Overall, 36.2% of Epromoters were shared between at least two cell lines (Figure 1E), supporting a physiologically diverse role of Epromoters.

We compared the average expression and tissue-specificity between non-redundant Epromoter-associated genes and the total set of genes using a comprehensive RNA-seq dataset across 30 tissues (32). We observed that Epromoter-associated genes were significantly more expressed (Figure 1F) and less tissue-specific (Figure 1G) than genes not associated with Epromoters. In order to compare our model Epromoters with a relevant set of typical promoters, we retrieved, for each of the 5743 Epromoters, a typical promoter associated with a gene with a matching expression pattern to the Epromoter-associated gene across different tissues (hereafter termed ‘control promoters'', n = 5743; Supplemental Figure S1B and C and ‘Materials and methods’ section). As shown in Figures 1F–G, genes associated with control promoters displayed similar average expression and tissue-specificity as Epromoter-associated genes, justifying the use of this control set as a proxy for typical promoters with similar promoter activity as Epromoters.

We assessed the transcriptional complexity of Epromoter-associated genes (i.e. number of TSS per gene) (Figure 1H). We observed that Epromoters were associated with genes harboring on average more TSS than other promoters (median value for Epromoters = 4). This suggests that in some cases, Epromoters might regulate an alternative promoter of the same gene, as previously suggested (6). We then assessed the 3D interactions between Epromoters and other distal promoters. We retrieved P–P interactions based on published promoter-capture HiC (24,34) and ABC models (35) across a wide set of tissues. We observed that Epromoters and control promoters displayed a higher number of promoter interactions as compared to typical promoters, and to a lesser extent, to control promoters (Figure 1I), supporting the idea that Epromoters are more likely to be involved in distal gene regulation.

Finally, we predicted that the inactivation of Epromoters should affect the expression of neighboring genes. To assess the impact of Epromoters on distal gene expression, we analyzed a comprehensive Perturb-seq dataset in which all coding-gene promoters had been repressed by CRISPRi followed by single-cell RNA-seq analysis (37). We first identified a set of 5054 promoters that had been efficiently inactivated (i.e. the associated gene is among the top 2 of repressed genes). We then identified the promoters for which CRISPRi resulted in the repression of *cis*-distal genes (<1 Mb) (Supplemental Table S4). We found that Epromoters significantly overlapped with the set of promoters associated with distal-gene regulation, while the control promoters did not (262 versus 205 for the Epromoters and control promoters, respectively; *P* value = 0.02, hypergeometric test). For example, CRISPRi repression of *DNAJC9-* and *ATP5MC1*-associated Epromoters resulted in the downregulation of P–P interacting genes *MRPS16* and *UBE2Z*, respectively (Figure 1J). Similar results were found using a CRISPRi screen with a more restricted dataset (38) (18 versus 11 for the Epromoters and control promoters, respectively; *P* value = 0.01, hypergeometric test). These results confirmed the potential regulation of distal genes by the identified Epromoters.

Overall, we have generated a comprehensive resource of human Epromoters based on STARR-seq data and confirmed their functional relevance as distal *cis*-regulatory elements.

### Epromoters display specific genomic and epigenomic features

We then asked whether there are specific genomic features that distinguish Epromoters from typical promoters. To make sure that the observed differences are not due to intrinsic promoter activity, but related to the enhancer activity, we further compared the Epromoters to the set of control promoters defined above.

**Figure 2.**
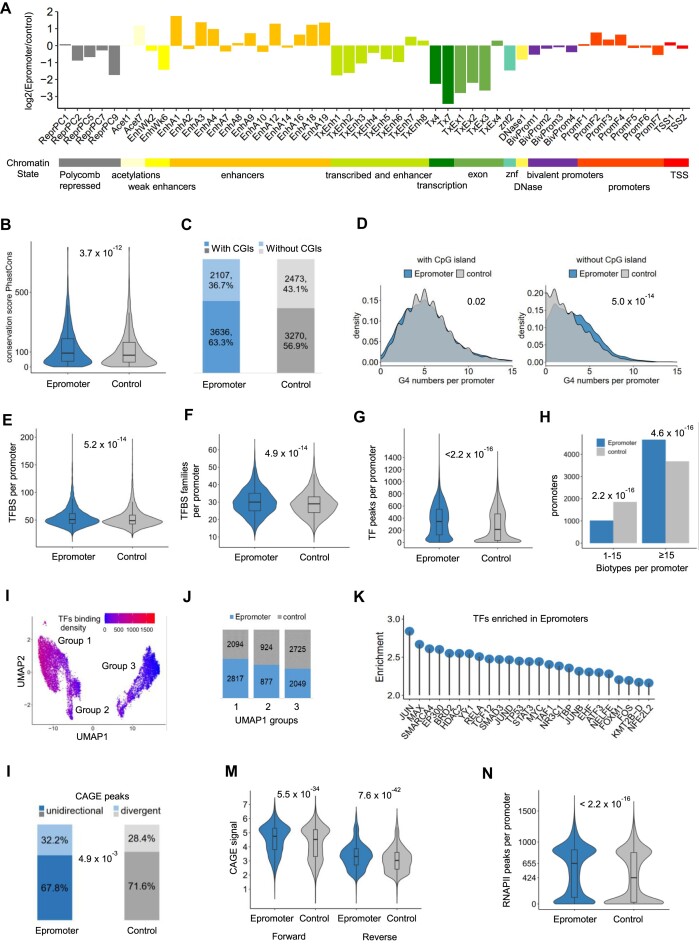
Epromoters display specific genomic/epigenomic features. (**A**) The chromatin state enriched at Epromoter and control promoters. The *Y*-axis represents the Log2-transformed fold change of the ChromHMM state enrichment ratio between Epromoter and control promoters (detailed in Supplementary Figure S2A). The bar colors at the bottom describes the group of ChromHMM states. (**B**) Conservation score of Epromoters and control promoters, which were retrieved from 470 mammalian species by PhastCons in UCSC genome browser. Statistical significance was assessed by a Wilcoxon test, and is represented by the *P*-value in the graph. (**C**) CGIs enriched in Epromoters. The bar plots show the percentage and number of Epromoters and control promoters with CGIs and without CGIs. Statistical significance was assessed by a Chi-squared test. (**D**) G4 numbers per promoter of Epromoters and control promoters with or without CGI. The density means the distribution of Epromoters or control promoters, which display the enrichment of Epromoters or control promoters. Statistical significance, as represented by the *P*-value in the graphs, was assessed by a Kolmogorov–Smirnov test. (**E** and **F**) Violin plots displaying the number of TFBS families per promoter (**E**; i.e. density) and the number of different TFBS families per promoter (**F**; i.e. diversity) using the JASPAR database. Statistical significance was assessed by a Wilcoxon test, and is represented by the *P*-value in the graphs. (**G**) Violin plots displaying the number of TF binding peaks per promoter identified by ChIP-seq using the ReMap resource. Statistical significance is represented by the *P*-value in the graph, as assessed by a Wilcoxon test. (**H**) Number of different tissues (Biotypes) of ChIP-seq peaks associated with Epromoters and control Epromoters as classified by ReMap. *P*-values are represented in the graph and were calculated by a Chi-squared test. (**I**) Dimension reduction by UMAP based on TF binding (ReMap) at each promoter. The color scale represents the TF binding density at each promoter. Three groups were manually separated based on the UMAP1 dimension. (**J**) Number of Epromoters and control promoters in each UMAP group defined in Figure 2I. (**K**) Top 25 TFs enriched at Epromoters, compared with control promoters. The height of the lollipop represents the odds ratio of TFs binding frequency between Epromoters and control promoters. (**L**) The percentage of unidirectional and divergent promoters as assessed by CAGE peaks. The *P*-value, as calculated by Chi-squared test, is represented in the graph. (**M**) Violin plots displaying the forward and reverse CAGE signal in function of the genomic orientation of the promoters. Statistical significance as assessed by a Wilcoxon test is represented by the *P*-value in the graph. (**N**) Violin plots displaying the number of RNAPII ChIP-seq peaks overlapping Epromoters and control promoters. Statistical significance, as assessed by a Wilcoxon test, is represented by the *P*-value in the graph.

First, we investigated the chromatin state of Epromoters and control promoters by using the full-stack ChromHMM model integrating over 1000 epigenome datasets (40). We found that Epromoters are relatively enriched for chromatin states related to active enhancers compared to control promoters (Figure 2A and Supplemental Figure S2A). Then we looked at the sequence conservation of Epromoters and control promoters by using different methods to calculate conservation scores, including PhyloP (42), PhastCons (43) and CADD scores (44). We found that Epromoters are significantly more conserved than control promoters (Figure 2B; Supplemental Figure S2B), potentially indicating that changes to Epromoters are unfavorable, i.e., they are likely to have an indispensable function.

CGIs are an important component of mammalian promoters. We found that 63% of Epromoters overlapped with CGIs as compared with 57% of control promoters (Figure 2C) (Chi-squared test, *P* value = 3.5 × 10^–12^), in agreement with the ubiquitous expression of Epromoter-associated genes. CGIs are naturally enriched for G-quadruplexes (G4), which are secondary DNA structures suggested to play an important role in defining the chromatin structure and regulatory activity of *cis*-regulatory elements (83–85). We, therefore, assessed whether G4 predictions were enriched at Epromoters (Figure 2D), using the G4hunter tool (46). While G4s were not enriched at Epromoters-overlapping CGIs, we found that non-CGI Epromoters harbor significantly more G4 as compared with control non-CGI promoters (Kolmogorov–Smirnov test). Similar results were obtained using different G4 prediction metrics (Supplemental Figure S2C and D). This suggests that beyond the CpG content, the density of G4 might have an important contribution to the Epromoter activity, reminiscent of a potential role of G4 structure at distal enhancers (86,87).

To assess the complexity of transcription factor binding sites (TFBS) in Epromoters compared to the control promoter set we retrieved the overlap between the family of TFBS (non-redundant) based on the JASPAR database (47) and promoter elements. We found that Epromoters displayed higher density (i.e. number of TFBS per promoter; Figure 2E) and diversity (i.e. number of different TFBS families per promoter; Figure 2F) of TFBS as compared to control promoters. We then assessed the number of different TF binding peaks (non-redundant) *per* promoter, using the ChIP-seq catalog from ReMap (48). We found that Epromoters were bound by a higher number of TFs (Figure 2G), and across a higher number of biotypes (i.e. different cell types; (Figure 2H) (Chi-squared test). These findings align with the understanding that Epromoters are more complex *cis*-regulatory elements and the broader expression of their associated genes. To assess how these properties are related to typical enhancers, we used a selection of enhancers based on the intersection between distal STARR-seq peaks and enhancer regions defined by the SCREEN database from ENCODE (53). We then compared the TFBS density and diversity (Supplementary Figure S3A and B) and the TF binding (Supplementary Figure S3C) of several properties between typical enhancers, Epromoters and control promoters. Interestingly, Epromoters displayed similar TFBS diversity compared to typical enhancers while displaying a significantly higher density of TFBS. In contrast, typical enhancers have relatively low TF binding compared to Epromoters and control promoters, likely reflecting that the formers are more tissue-specific.

To determine whether TF binding could distinguish between Epromoters and typical promoters, we conducted a nonlinear dimensionality reduction using UMAP analysis using the TF binding information from ReMap (Figure 2I). We found that the primary dimension (UMAP1) was tightly associated with TFBS density (Figure 2I). Strikingly, three promoter groups could be identified based on the UMAP1 dimension that roughly separated Epromoters from control promoters (Figure 2J; *P* value = 1.1 × 10^–45^; Chi-Squared test comparing group 1 versus group 3; Supplemental Figure S2E), with the Epromoter-enriched cluster (group 1) displaying higher TF binding density. We then identified the TFs that were specifically enriched in Epromoters as compared to control promoters (Figure 2K). Among the top 25 enriched TFs, we found several inducible TFs such as the AP1 family (JUN, JUND and FOS), NfkB (RELA), STAT3 and ATF3. Strikingly, amongst the top 5 enriched TFs, we found three general co-factors associated with enhancer function (Supplemental Figure S2F–H). These included the EP300 histone acetylase, which is a hallmark of active enhancers (88); the ATP-dependent chromatin remodeler SMARCA4, which is associated with accessibility landscape of tissue-unrestricted enhancers (89) and the bromodomain-containing protein 2 (BRD2), which is associated with the chromatin insulator CTCF and the cohesin complex to support *cis-*regulatory enhancer assembly for gene transcriptional activation (90).

Finally, we investigated the association with transcription initiation using the CAGE resource from FANTOM5 (49). It has been previously shown that the strength of transcription initiation correlated with enhancer activity at both proximal and distal regulatory elements (6,16,91–93). We observed that Epromoters were more frequently associated with divergent transcription (Figure 2L; Chi-squared test, *P* value = 4.9 × 10^–3^), as well as with more forward and reverse CAGE signals (Figure 2M). This latter observation suggests that Epromoters are associated with increased (bidirectional) transcription initiation, potentially reflecting the enhancer activity of Epromoters. Additionally, we assessed the recruitment of RNA-Polymerase II (RNAPII) to Epromoters and controls using a comprehensive RNAPII binding atlas (52). Consistent with the CAGE results, we observed that Epromoters display more RNAPII binding (Figure 2N). This is reminiscent of a recent study suggesting a prominent role of RNAPII binding on the stabilization of distal interaction between *cis*-regulatory elements (94).

Overall, we found that Epromoters display specific genomics and chromatin features compared to control promoters with similar transcriptional activity.

### Genetic variation associated with Epromoters.

**Figure 3.**
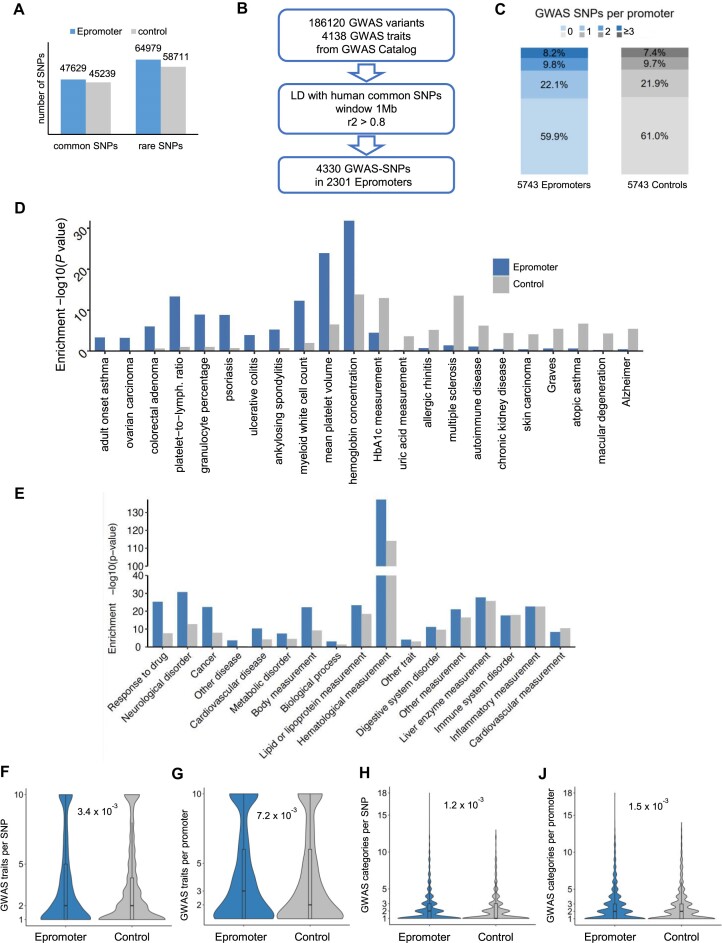
Genetic variation associated with Epromoters. (**A**) Number of common and rare SNPs overlapped with Epromoters and control promoters. (**B**) Scheme to identify GWAS-SNPs in Epromoters. First, 186120 tag SNPs associated with 4138 GWAS traits were collected from the GWAS Catalog. The SNPs from the GWAS Catalog were linked with common SNPs by a stringent linkage disequilibrium (LD) threshold (r2 > 0.8) within 1 Mb. Then the LD SNPs associated with GWAS (GWAS-SNPs) were overlapped with Epromoters. Finally, 4330 GWAS-SNPs were found in 2301 Epromoters. (**C**) Distribution of GWAS-SNPs per Epromoters or control promoters. (**D**) GWAS traits differentially enriched in Epromoters. The GWAS trait enrichment was calculated by the ratio of SNPs associating each GWAS trait between Epromoters or control promoters *versus* the whole genome. The *P* values for enrichment were calculated by the hypergeometric test. Only differentially enriched GWAS traits between Epromoters and control promoters and associated with a known GWAS category are shown in the plot. Statistical significance for the difference was assessed by the Chi-squared test. (**E**) GWAS categories enriched in Epromoter and control promoters. The GWAS category enrichment was calculated by the ratio of SNPs associating each GWAS category between Epromoters or control promoters *versus* the whole genome. The *P* values for enrichment were calculated by the hypergeometric test. (**F** and **G**) Violin plots displaying the number of GWAS traits *per* SNP (**F**) and promoter (**G**). Statistical significance, as assessed by a Wilcoxon test, is represented by the *P*-value in the graph. (H and I) Violin plots displaying the number of GWAS categories per SNP (**H**) and per promoter (**I**). Statistical significance, as assessed by a Wilcoxon test, is represented by the *P*-value in the graph.

First, we overlapped Epromoters and control promoters with both rare and common variants (SNP) from the SNPdb (NCBI) database. Both common and rare variants were significantly enriched at Epromoters (*P* values = 4.7 × 10^–15^ and 6.2 × 10^–71^, respectively; Chi-Squared test). We then extracted 186120 variants associated with 4138 GWAS from the GWAS catalog (54) and retrieved over 2.4 million common SNPs in high linkage disequilibrium (LD; r^2^ > 0.8; 1000 Genomes project) with GWAS tag SNPs (Figure 3B; hereafter, GWAS-SNPs). We obtained 4330 and 4062 GWAS-SNPs overlapping 2301 Epromoters and 2241 control promoters, respectively (Supplemental Table S5). In fact, 40% of Epromoters and 39% of control promoters harbored at least one GWAS-SNP (Figure 3C).

We further investigated the enrichment of GWAS in Epromoters. In total, 1251 GWAS traits are associated with 4330 SNPs at Epromoters (Supplemental Table S5). We found that 184 GWAS traits were significantly enriched at Epromoters compared to the genome background (*P* value < 0.001; hypergeometric test) while 12 GWAS traits were differentially enriched as compared with control promoters (Figure 3D; *P* value < 0.05; Chi-squared test; Supplemental Table S6). We classified the GWAS traits into 17 categories, as defined by the EFO (Experimental Factor Ontology) database (57), and compared the relative enrichment between Epromoters and control promoters (Figure 3E). We found specific Epromoter enrichment for certain categories, including disease-related categories such as neurological disorders, cancer, and metabolic diseases, as well as response to drugs. To assess the heritability of Epromoters for GWAS, we calculated the partitioned heritability of 176 GWAS summary statistics using the LD score regression model (58) for Epromoters, control promoters, FANTOM-enhancers (49) and UCSC-defined promoters and coding regions (Supplemental Figure S4). We observed that certain GWAS traits displayed high heritability either in Epromoters, control promoters, or enhancers, but low heritability in total promoters and coding regions. Overall, we found that Epromoters were associated with specific physiological traits or diseases.

Further analysis focused on the association of Epromoters with multiple GWAS traits. Epromoters exhibited significantly more GWAS traits *per* GWAS-SNP and *per* promoter than control promoters (Figure 3F and G, respectively), although the effect size was relatively small. This observation suggested that Epromoters are associated with a broader range of traits, possibly indicating pleiotropy, referred here as to a single *cis*-regulatory element affecting more than one trait independently (95). Additionally, we investigated whether pleiotropic GWAS-SNPs were associated with different GWAS categories. Indeed, Epromoters and their associated GWAS-SNPs were found to be more frequently associated with different GWAS categories (Figure 3H and I, respectively), supporting the hypothesis that Epromoters play a more pleiotropic role than typical promoters in influencing diverse traits. Similar analyses performed with only disease-associated categories also demonstrated a higher pleiotropy of Epromoters compared to control promoters (Supplemental Figure S5A–D), suggesting that our observation did not depend on potential bias in the choice of traits for GWAS. Pleiotropy has previously been linked to the breadth of gene expression (96). Although Epromoters and control promoters are associated with genes with similar tissue-specificity (Figure 1G), we assessed whether the observed pleiotropy might be associated with the degree of tissue-specificity. To this aim, we compared the extent of pleiotropy between three categories of promoters associated with different degrees of tissue specificity. As expected, ubiquitous promoters demonstrated higher levels of pleiotropy compared to tissue-specific promoters (Supplemental Figure S5E). Interestingly, however, while the most ubiquitous and tissue-specific categories (specificity scores [0–0.25] and [0.75–1], respectively) did not show significant differences between Epromoters and control promoters, the intermediate category (specificity scores [0.25–0.75]), representing genes of which the expression is regulated across multiple tissues, demonstrated a greater extent of pleiotropy in the case of Epromoters, suggesting that the specific pleiotropic association of Epromoters depends on the breadth of gene expression. To assess whether the higher pleiotropy observed at Epromoters was sensitive to the window size used to define the promoter region, we performed a similar GWAS analysis using a window of 250 bp (instead of 500 bp) and obtained consistent results (Supplementary Figure S5F–I). To note, both Epromoters and control promoters displayed higher pleiotropy than distal enhancers, likely due to the more ubiquitous activity of these promoters (Supplemental Figure S3D and E).

Finally, we aimed to validate our observations by analyzing a dataset linking pleiotropy with genetic architecture in complex traits (96). The study provided statistical association of individual SNPs with either single domains (i.e. associated with one or more traits from a single domain) or multi-domain (i.e. associated with traits from multiple domains). Using this dataset, we observed that the Epromoter set was associated with a higher number of multi-domain SNPs (Supplemental Figure S6; *P* value = 0.03, Chi-squared test), independently confirming the high level of pleiotropy found at Epromoters. Overall, our results suggested that Epromoters have a higher tendency to be associated with multiple GWAS traits, and we hypothesized that this pleiotropy might be due to the regulation of multiple genes by the Epromoters.

### Epromoter's pleiotropy is associated with the regulation of multiple target genes

**Figure 4.**
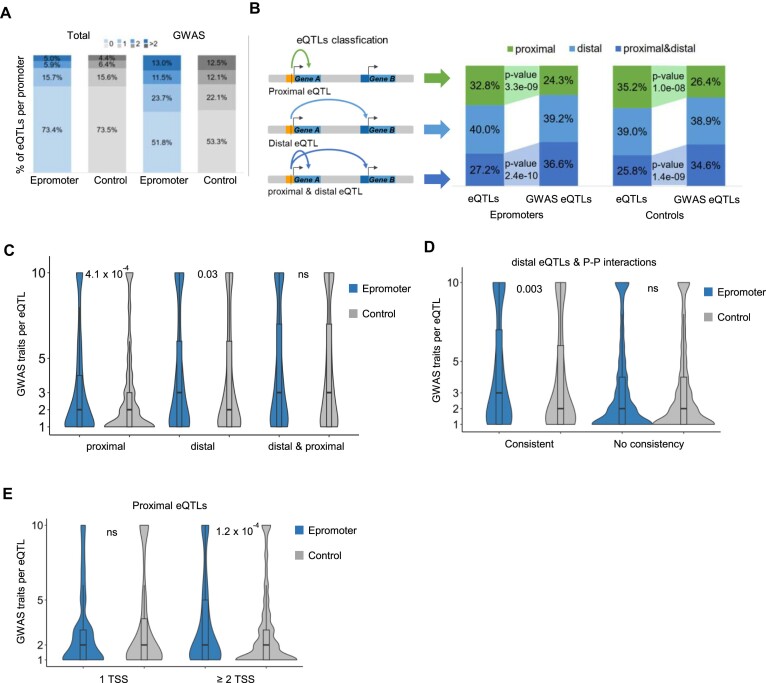
The link between pleiotropy and target genes. (**A**) Percentages of Epromoters and control promoters according to the number of eQTLs per promoter and considering either all SNPs or only the GWAS-associated SNPs. (**B**) The eQTLs were classified into proximal, distal or proximal & distal eQTLs as indicated in the left panel. The right panels indicate the percentages of promoters associated with the different types of eQTLs. P-values were calculated by a Chi-Squared test. (**C–E**) Violin plots displaying the number of GWAS traits per eQTL in the function of the eQTL type (**C**), eQTLs with distal targets consistent or inconsistent with P–P interactions (**D**), and the number of TSS per gene associated with proximal eQTLs (**E**). Statistical significance between Epromoter and control sets was assessed by a Wilcoxon test, and is represented by the *P*-value in the graph.

To identify potential target genes associated with Epromoter variation, we integrated expression eQTL datasets obtained from the fine-mapped credible sets within the EBI eQTL Catalog (59). From 9137260 fine-mapped eQTLs, we found 5843 associated with 2768 Epromoters and 5644 associated with 2684 control promoters (Supplemental Table S5). In general, Epromoter and control promoter-associated GWAS-SNPs were found to be enriched in eQTLs (Figure 4A) (*P* values = 6.2 × 10^–59^ and 2.6 × 10^–53^ for Epromoter and control sets, respectively; Chi-squared test), highlighting the regulatory potential of these variants. Specifically, 48.2% and 47,7% of GWAS-associated Epromoters and control promoters overlapped with at least one eQTL, respectively. Among 2768 Epromoters and 2684 control promoters with eQTLs, approximately half exhibited at least two eQTLs.

Furthermore, our analysis delved into the association of eQTLs with proximal, distal, or both proximal and distal target genes (Figure 4B). As expected, Epromoter eQTLs were less associated with proximal genes as compared with control eQTLs (Figure 4B) (*P* value = 0.006 for all eQTLs, *P* value = 0.05 for GWAS eQTLs, Chi-squared test). Surprisingly, GWAS SNPs were depleted of proximal eQTLs (*P* value = 3.3 × 10^–9^, Wilcoxon test) and enriched in proximal-distal eQTLs (*P* value = 1.4 × 10^–9^, Wilcoxon test) for both Epromoter and control promoters sets. However, this category might represent a mixture of *cis* and *trans* effects (97–99), likely combining a *cis* effect on the proximal gene and trans effects on distal genes (see below).

Next, we compared the pleiotropic impact on diseases of eQTLs associated with either proximal, distal or proximal and distal genes (Figure 4C). On the one hand, both Epromoters and control eQTLs with both proximal and distal targets were highly pleiotropic, with no significant differences between the two sets (median of GWAS traits = 3; Wilcoxon test). As mentioned above, we believe most of these pleiotropic eQTLs are associated with both *cis* and *trans* effects. On the other hand, Epromoters with proximal- or distal-only eQTLs demonstrated higher pleiotropy than corresponding control eQTLs, suggesting a stronger role of Epromoter variants on *cis*-regulatory functions. To confirm that the pleiotropy associated with distal eQTLs from Epromoters was due to *cis* interaction with distal targets (as opposed to *trans* effects), we analyzed their consistency with P–P interactions (Figure 4D). Strikingly, Epromoters with consistent distal targets displayed a significant increase in pleiotropy, affirming the link between Epromoter variants and the actual regulation of distal genes. Control promoters did not exhibit the same trend, emphasizing the unique regulatory role of Epromoters in distal gene interactions.

As we found that Epromoters are frequently associated with genes harboring multiple TSSs (Figure 1H), we wondered whether the higher pleiotropy observed with proximal eQTLs might be linked to distal regulation by alternative promoters as previously suggested (6). Indeed, we observed a higher pleiotropy only at Epromoters associated with multiple TSSs (Figure 4E). In fine, the higher pleiotropy observed at Epromoters appears to be linked to the actual regulation of distal targets, including either alternative promoters or distal genes.

### A pleiotropic Epromoter variant associated with COVID-19 shows enhancer/promoter switch

**Figure 5.**
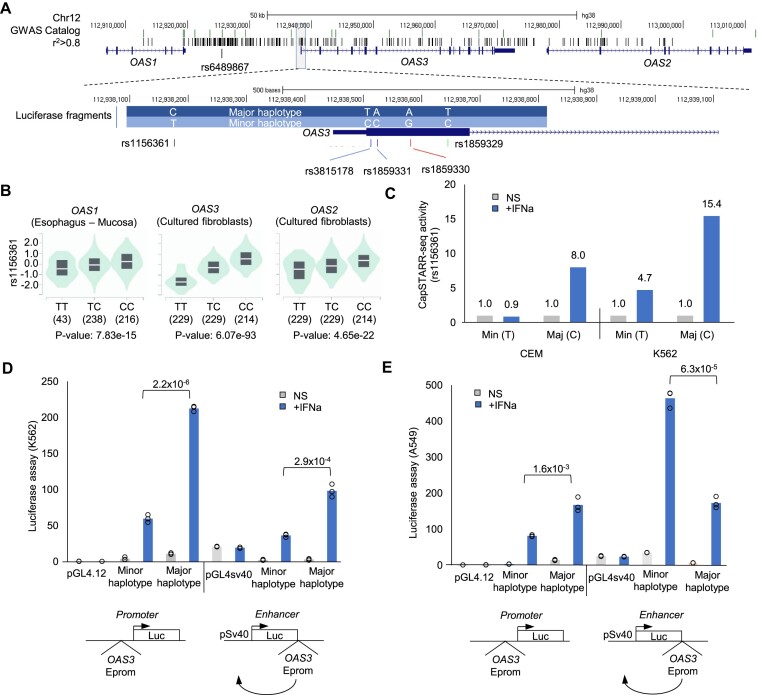
A pleiotropic Epromoter variant associated with COVID-19 shows enhancer/promoter switch (**A**) UCSC browser view of OAS1/2/3 locus, with lead COVID-19 SNP rs6489867 and SNPs in LD (r^2^> 0.8), as well as the location of the 726 bp region containing 5 SNPs in the OAS3 Epromoter analyzed in Figure 5D and E. (**B**) eQTL (GTEx) of rs1156361, showing decreased expression of the OAS1/2/3 of the minor allele. (**C**) CapSTARR-seq activity of the OAS3 Epromoter containing the rs1156361 minor (Min T) or major (Maj C) alleles in the CCRF-CEM and K562 cell lines with no stimulation (NS) and with 6 h of IFNa stimulation showing increased regulatory activity of the major allele upon IFNa stimulation as compared to the minor allele in both cell lines. (**D** and **E**) Luciferase reporter assays assessing the promoter (left panel) or enhancer (right panel) activity of the OAS3 Epromoter harboring the minor or major haplotypes before and after IFNa stimulation for 6 h in the K562 (**D**) and A549 (**E**) cell lines. Luciferase experiments were performed in triplicate and statistical significance was assessed by Students’ t-test, as represented by the *P*-values in the graphs.

Among the promoters that contain disease-associated SNPs, we identified six Epromoters that we previously demonstrated by CRISPR-Cas9 genetic deletion to regulate distal genes, including *OAS3*, *ISG15, IFIT3, IL15R, METTL21* and *BAZ2B* (6,9) (Supplemental Table S5). This supported a functional link between the genetic variants at these Epromoters and the regulation of distal genes. Among those, the OAS3 Epromoter provided a remarkable example of a pleiotropic locus. The *OAS3* gene is embedded in a cluster that also includes *OAS1* and *OAS2* (Figure 5A), which all encode for the oligoadenylate synthetase (OAS) family of proteins and play an important role in antiviral immunity (100). The *OAS1/2/3* locus is a highly pleiotropic locus associated with several diseases, including asthma, blood protein measurement, chronic leukemia, systemic lupus erythematosus and severe COVID-19 (Supplementary Table S5). Furthermore, the minor allele haplotype of the *OAS1/2/3* locus is a Neanderthal haplotype, first introduced into the modern human population by interbreeding with Neanderthals around 50 000 years ago (101). This haplotype spans a 75-kb region, and variants of this haplotype have been associated with protection against West Nile Virus (102), increased resistance to hepatitis C infection (103), and protection against SARS-CoV (He at al 2006), and most recently with reduced risk of becoming severely ill upon SARS-CoV-2 infection (104,105). The *OAS3* promoter showed IFNa-dependent enhancer activity in Hela, K562 and CCRF-CEM cell lines (Supplementary Table S3). Strikingly, we previously showed that deletion of the *OAS3* Epromoter resulted in impaired induction of the entire *OAS* cluster after IFNa stimulation (9), suggesting this element is a master regulator of the interferon response of the locus. Since there is no indication of other regulatory regions within the *OAS1/2/3* locus, except the promoters of the three genes (9), we assumed that *cis*-regulatory variants mainly reside in the *OAS3* Epromoter.

We initially identified rs1156361 (located 352 bp upstream of the *OAS3* TSS) as a GWAS-SNP within the *OAS3* Epromoter (Supplementary Table S5). eQTL data of the GTEx database indicates that the minor allele of rs1156361 is associated with lower expression of all three *OAS* genes in multiple tissues (Figure 5B), consistent with the role of this Epromoter as a master regulator of the *OAS* locus. We realized that the promoter library used for the CapSTARR-seq experiments contains both alleles of the rs1156361 SNP. We therefore assessed the allele-specific activity of this SNP in the K562 and CCRF-CEM cell lines with or without IFNa stimulation (Figure 5C). We observed that the *OAS3* Epromoter harboring the major allele (C) displayed a significantly higher enhancer activity upon stimulation with IFNa. Upon closer inspection of the *OAS3* promoter [-500 bp; 250 bp], we found 4 SNPs in high LD (r^2^> 0.97 in the European population) with rs1156361: rs3815178 and rs1859331 (5′ UTR variants), rs1859330 (missense variant) and rs1859329 (synonymous variant). These additional 4 SNPs are also in eQTLs with *OAS1/2/3* with the same directionality as rs1156361 (Supplemental Figure S7). To assess the contribution of the two haplotypes on the relative promoter and enhancer activity of the *OAS3* Epromoter, we performed luciferase reporter assays in K562 cells using a 726 bp genomic region containing the five SNPs. We observed that the major haplotype confers both a stronger promoter and enhancer activity in K562 after IFNa stimulation (Figure 5D). We also performed the luciferase reporter assays in A549 cells, a lung epithelial cell line commonly used as a model for COVID-19 (106–108). In this cell line, the major haplotype similarly conferred stronger promoter activity, but the minor haplotype displayed stronger enhancer activity (Figure 5E). Interestingly, there was a higher absolute promoter activity in IFNa-treated K562 cells compared to the enhancer activity, while the opposite was observed in A549 cells. Overall, these results suggest that the pleiotropic association of the *OAS1/2/3* locus with multiple diseases, including severe COVID-19, might be explained, at least partially, by transcriptional deregulation of all three *OAS* genes by regulatory variants lying within the *OAS3* Epromoter. Our results also highlight the differential impact of genetic variants on enhancer versus promoter activity of Epromoters.

### Functional assessment of pleiotropic Epromoter variants

**Figure 6.**
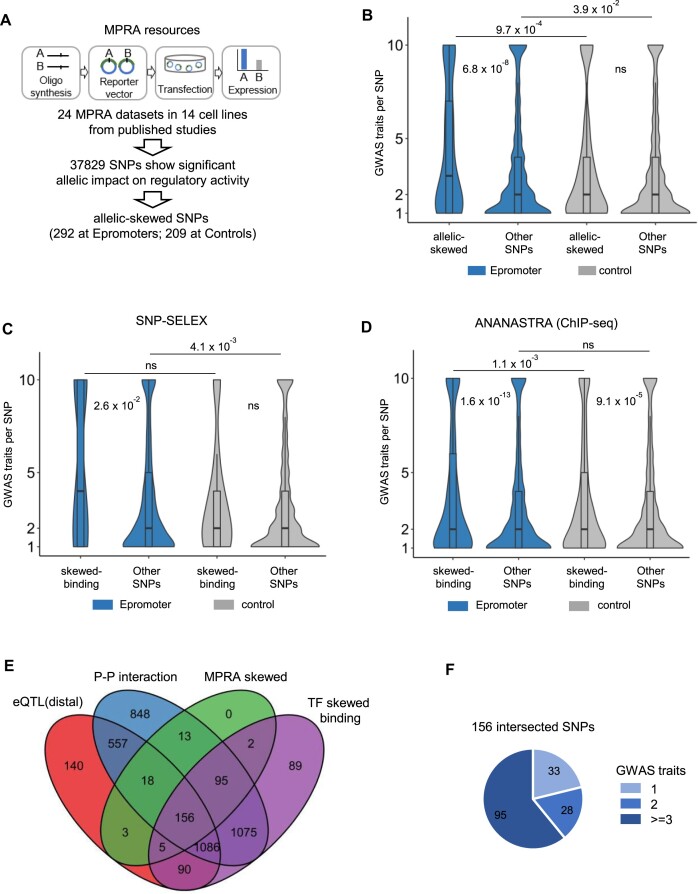
Functional validation of pleiotropic Epromoter SNPs. (**A**) Schematic strategy to identify GWAS-SNPs with allelic-skewed regulatory activity. First, 24 MPRA datasets in 14 cell lines were collected from published studies. Around 37 831 SNPs in total show a significant allelic impact on regulatory activity. Finally, 292 allelic-skewed SNPs were overlapped within Epromoters. (**B**) Violin plots displaying the number of GWAS traits per SNP in the function of whether the SNP had an allelic-skewed regulatory activity or not (other SNPs) based on MPRA experiments. Statistical significance was assessed by a Wilcoxon test, and is represented by the *P*-values in the graph. (**C-D**) Violin plots displaying the number of GWAS traits per SNP in the function of whether the SNP had a skewed TF binding based on SNP-SELEX assays (**C**) and ANANASTRA (**D**). Statistical significance was assessed by a Wilcoxon test, and is represented by the *P*-values in the graphs. (**E**) The Venn diagram illustrates the intersections of SNPs located at Epromoters among four categories: eQTLs with distal effects, P–P interactions, allelic-skewed SNPs identified by MPRA, and SNPs exhibiting skewed TF binding. (**F**) The pie chart shows the number of non-pleiotropic (1 GWAS trait) and pleiotropic (≥2 GWAS traits) SNPs from the 156 intersected SNPs.

To globally assess the functional impact of Epromoter's variants, we compiled the results from 24 published Massive Paralleled Reporter Assays (MPRA) experiments (Supplemental Table S7), which have assessed the regulatory impact of genetic variants. From 37829 SNPs with significant allelic impact on regulatory activity (allelic-skewed SNPs), 292 and 209 overlapped with GWAS-SNPs from Epromoter and control promoters, respectively (Figure 6A). Strikingly, Epromoter GWAS-SNPs with MPRA-validated allelic impact displayed significantly higher pleiotropy (Figure 6B), while control GWAS-SNPs did not. To further explore the functional relevance of Epromoter GWAS-SNPs, we assessed the impact on TF binding by interrogating the SNP-SELEX dataset (78), which systematically assessed the binding of 270 human TFs to 95886 noncoding variants in the human genome using an ultra-high-throughput multiplex protein-DNA binding assay (Figure 6C). We found that Epromoter GWAS-SNPs that impact TF binding (skewed TF binding) displayed higher pleiotropy than the remaining Epromoter GWAS-SNPs, while there were no significant differences in the case of control promoters. Similar results were observed when analyzing allelic-specific TF binding *in vivo* using the ANANASTRA resource (79) (Figure 6D). Altogether, these results suggest that the observed pleiotropic effects are due to the functional impact of Epromoter's variants in terms of skewed *cis*-regulatory activity and TF binding.

**Figure 7.**
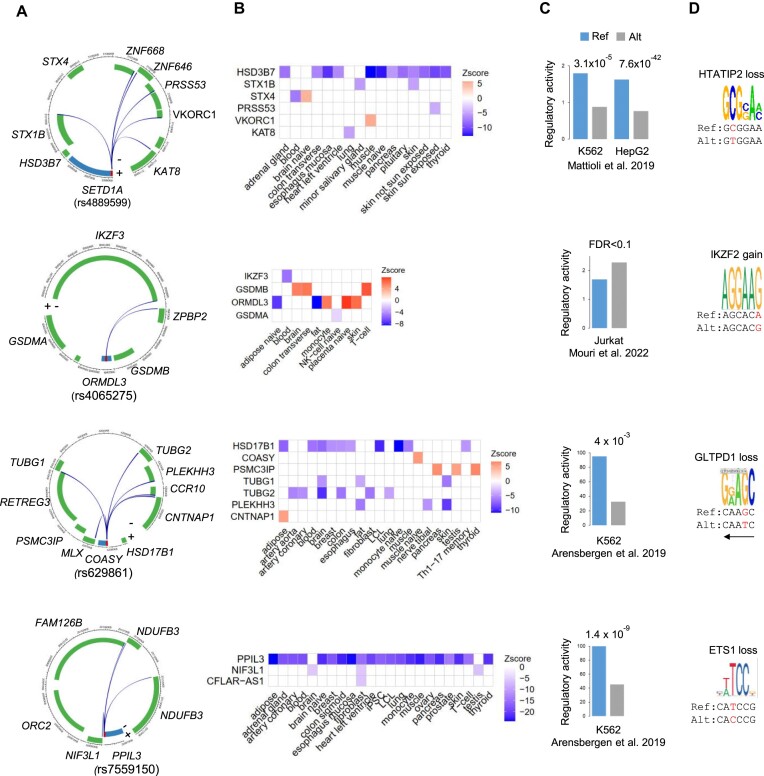
Examples of pleiotropic Epromoters (**A**) The circular visualization of Epromoters and interacting genes based on their genomic locations. The Epromoter-associated genes are displayed in blue, while the red bar represents the Epromoters. Genes in the outer circle are in the positive strand. Genes in the inner circle are in the negative strand. The selected SNP is indicated under the Epromoter-gene name. The curves are P–P interactions. (**B**) The heatmaps show the eQTLs effect of the selected SNPs on target genes in different tissues from the eQTL Catalogue. Each row in the heatmap represents the gene associated with the eQTL. Each column represents the tissue of eQTL. The color scale represents the z-score of the eQTL effect on target genes. (**C**) The bar plots show the allelic-skewed regulatory activity of the selected SNPs validated by MPRA. *P*-values or FDR according to original studies are shown at the top of the graphs. (**D**) Representative TFBSs affected by the selected SNPs. The predicted consequences of the SNPs (from reference to alternative alleles) are shown at the top. The sequences of reference and alternative alleles are shown at the bottom. The SNP is highlighted in red. The arrow indicates the sequence is in the reverse complement.

We next integrated the different levels of validation resources to retrieve a list of 156 Epromoter overlapping GWAS-SNPs with consistent distal eQTLs, P-P interactions, allelic-skewed MPRA activity and TF binding (Figure 6E). From this list, 28 (18.4%) SNPs were associated with 2 traits and 95 (62.5%) SNPs were associated with three or more traits, thus representing a resource of *bona fide* pleiotropic Epromoters (Figure 6F; Supplemental Table S5). Figure 7 provides four examples of pleiotropic Epromoters (*SETD1A, COASY, ORMDL3* and *PPIL3*) with consistent 3D interaction and eQTL target genes (Figure 7A and B), significant differences on allelic regulatory activity (Figure 7C) and predicted perturbation of TF binding (Figure 7D; Supplemental Table S5). Careful examination of proximal and distal target genes suggested that the association with multiple GWAS traits might be explained by the combination of the individual gene functions (see Supplementary Information 1 for a detailed description of each locus). For example, The *SETD1A* Epromoter is a highly pleiotropic locus involved in over 30 diverse GWAS traits, including immune-associated diseases (Graves, psoriasis, Crohn's, eosinophil count), neurological diseases (Parkinson's, epilepsy, anxiety) and heart disease risk factors (BMI, blood and pulse pressure, triglycerides and LDL cholesterol measurements). The associated rs4889599 SNP displayed allelic-skewed MPRA activity (Figure 7C, top panel) and is predicted to affect the binding of the HTATIP2 (Figure 7D, top panel; Supplementary Table S5). Interestingly, *SETD1A* and the *STX1B* distal target are both associated with neurological disorders, while the *HSD3DB7* and *STX4* distal targets are associated with immune-related and cardiometabolic diseases, respectively (Supplementary Information 1). Overall, we concluded that the pleiotropic association of Epromoters with multiple diseases and traits is linked to the *cis*-regulatory impact of the genetic variants and the combination of the physiological functions of proximal and distal target genes.

## Discussion

Genome-wide studies have become pivotal in unraveling the genetic basis of complex traits through the identification of SNPs associated with specific phenotypes. In this study, we employed a comprehensive approach to investigate the genetic landscape of Epromoters, an unconventional type of *cis*-regulatory element harboring both enhancer and promoter functions. We first demonstrated that Epromoters display distinct genomic and epigenomic features compared to typical promoters that harbor similar promoter, but not enhancer, activity. We then examined their association with genetic variants, particularly focusing on SNPs identified in GWAS. Our comprehensive analysis provides novel insights into the genetic variation within Epromoters, highlighting their potential roles in complex trait regulation. The enrichment of specific GWAS traits and the increased pleiotropy observed in Epromoters, as compared with control promoters, highlighted their importance in the genetic architecture of complex traits and diseases. Although the overall pleiotropic effect of Epromoters is rather small compared to control promoters, we observed that these differences are maintained when considering only disease-related traits or when accounting for gene-expression breadth. Moreover, the consistency between eQTLs and 3D interactions (Figure 4D) or the experimentally validated GWAS SNPs (Figure 7B and C) demonstrated a stronger and more specific pleiotropy at Epromoters, supporting the hypothesis that the observed pleiotropic effects are likely due to the functional impact of Epromoter variants on distal genes. Our findings underscore the intricate relationship between Epromoter-associated genetic variation, eQTLs, and pleiotropy, unraveling the potential regulatory impact on both proximal and distal target genes. The identified link between Epromoters and distal gene regulation provides valuable insights into the functional genomics of complex traits and paves the way for a deeper understanding of the molecular mechanisms underlying pleiotropy.

A major paradigm in the field of gene regulation is to understand what are the molecular bases of proximal (promoter) *versus* distal (enhancer) functions (12). Although a unified model of *cis*-regulatory functions has been proposed (16), several studies, including ours, have suggested that intrinsic (binding sites, nucleotide composition, etc.) and extrinsic (TFs, genomic context, etc.) features that drive enhancer and promoter activities are not the same (4,6,9,10,16,91–93). Previous studies have shown that the type of TF that binds a *cis*-regulatory element might influence the relative enhancer or promoter activity (4,12). Similarly, we showed that interferon-response Epromoters have a higher density and better quality of interferon-stimulated response elements, as compared with typically induced promoters, which, in turn, results in the Epromoter-specific recruitment of STAT1/2 and IRF TFs and activation of neighbor genes (9).

Here, we took advantage of the comprehensive Epromoter resource we have built to perform a thorough comparison of Epromoters against typical promoters displaying similar promoter activity. Our results revealed several intrinsic differences between Epromoters and typical promoters. First, Epromoters are associated with genes that are less tissue-specific and harbor multiple alternative promoters. Second, they are involved in a higher number of interactions with other promoters. Third, their sequences are more conserved and display a higher number of G4 elements. Fourth, Epromoters have a higher density and diversity of TFBSs, which is reflected by a high density of TF binding. Interestingly, the high diversity of TFBS is a common feature of Epromoters and distal enhancers, while the high density of TFBS appears to be a specific feature of Epromoters. Finally, Epromoters display a higher level of sense and antisense transcription initiation which is reflected by a higher overlap with RNAPII binding. Based on these findings, we speculate that Epromoters represent a combination of the two types of *cis*-regulatory elements, thus combining features associated with enhancer and promoter activities within an enhancer-promoter continuum of *cis*-regulatory elements. This intermediated position implies that Epromoters might display a higher density and complexity of TFBS because it has to accommodate the binding of TFs for both enhancer and promoter functions. In this scenario, typical promoters are enriched in binding sites for TFs conferring promoter activity and enhancers enriched in binding sites for TFs conferring enhancer activity, while Epromoters are enriched for both types of binding sites leading to a higher density of TFBS. Future works should systematically assess the contribution of TFBS and associated TFs to the enhancer and promoter activity in order to better understand the molecular features that determine the intrinsic promoter and enhancer potentials of *cis*-regulatory elements, and in particular of Epromoters. This, in turn, might help to better predict the impact of mutations or natural variants of Epromoters that might affect either proximal or distal gene regulation.

Several studies, including ours, have demonstrated that human genetic variation within Epromoters influences distal gene expression (6,17,23–25). Moreover, specific examples highlight the distal impact of disease-associated variants within Epromoters (10,26,109–114). The complex regulation by Epromoters might therefore have two predicted consequences. On the one hand, there might be a general underestimation of the impact of Epromoter variation in disease because the causal gene might not be the closest one and therefore the link between genotype and phenotype might be missed in many case studies. On the other hand, as Epromoters potentially control several genes at the same time and efficiently recruit key TFs, mutations in these regulatory elements are expected to have a stronger pathological impact, as compared to typical promoters. This might result from the regulation of multiple genes either involved in the same (additive or synergistic effects) or different (pleiotropy) pathways. Indeed, our present work reveals that genetic variants within Epromoters linked to GWAS are significantly associated with multiple diseases as compared with typical promoters, supporting the hypothesis whereby Epromoters might have a pleiotropic effect in disease by perturbing the expression of several genes at the same time.

Pleiotropy, implying a single *cis*-regulatory element affecting more than one trait independently, could be due to the perturbation of a single gene playing multiple functions in different tissues (115,116) or the regulation of multiple genes in the same or different tissues (117,118). Our results rather point to the latter possibility. On the one hand, we observed that pleiotropy is associated with an increased number of target genes, as assessed by consistent eQTL and P–P interactions. While it is difficult to ensure that all Epromoter variants are *bona fide* distal regulators, we noticed that taking into consideration functional assessment of allelic-specific activity by MPRA allows for significant enrichment of pleiotropic Epromoters. On the other hand, a careful examination of several pleiotropic Epromoters, reveals that the different target genes play a role in different physiological functions that might explain the association with the different diseases. Future work will require extensive functional studies of the target genes in order to demonstrate their involvement in the context of each associated disease. In line with our finding, a schizophrenia-risk SNP within the promoter of the *VSP45* gene was shown to *cis*-regulate three genes via allele-specific chromatin looping. These genes act in a non-additive synergistic fashion to enhance dendritic complexity and neuronal activity (119). In conclusion, genetic alterations affecting Epromoters are likely to have a stronger impact on the regulation of disease-associated genes, as compared with typical promoters.

The regulation of the *OAS1/2/3* locus by the *OAS3* Epromoter provides a detailed illustration of the link between Epromoters and pleiotropic association with diseases. In particular, the expression of *OAS1/2/3* has been associated with severe COVID-9 (105,120–122). Previous studies have described COVID-19-associated variants linked to either *OAS1* (123) or *OAS2* (124) expression, but failed to explain the global deregulation of the entire cluster. Although we cannot exclude the existence of multiple causal variants working synergistically, our results provide a more straightforward mechanistic explanation for the deregulation of the entire cluster, whereby the Neandertal inherited *OAS3* Epromoter haplotype (minor allele) displays lower promoter and enhancer activity, therefore resulting in decreased expression of the three *OAS* genes. However, the relative enhancer/promoter switch observed in the A549 cell line might indicate a more complex regulatory network that will need to be explored in the future.

Genetic variation might impact the expression of neighboring genes in the same (e.g. enhancer and promoter activity are equally affected) or opposite (e.g. the genetic variant induces an enhancer/promoter switch) directions. For instance, two studies demonstrated that an alternative variant associated with prostate cancer increases the enhancer activity of the promoter leading to decreased expression of the proximal transcript but increased expression of two distal transcripts directly involved in cancer progression (113,114). Moreover, the genetic variants might differently impact enhancer and/or promoter activity in different tissues. For instance, Leung et al. found frequent examples of dynamic epigenetic switches where active promoters in one tissue displayed a histone modification signature of enhancers in other tissues/cell types (27). Similarly, Chandra et al. found a substantial number of P–P interactions involving transcriptionally inactive genes, suggesting that non-transcribing promoters may function as active enhancers for distal genes (26). An enlightening example is provided by the *OAS1/2/3* locus, where genetic variation at the *OAS3* Epromoter affects IFNa-dependent enhancer and promoter activity in both K562 and A549 cell lines. However, the relative impact on enhancer and promoter activities switches between the two cell lines.

Overall, by leveraging extensive genomic and functional datasets, our study explores the intricate relationship between Epromoter variation, pleiotropy and target gene regulation, shedding light on the complex regulatory mechanisms underlying the genetic architecture of complex traits.

## Supplementary Material

gkae1270_Supplemental_Files

## Data Availability

The raw sequencing data and processed files of CapSTARR-seq generated in this study have been submitted to the Gene Expression Omnibus (GEO) under the accession GSE268615. All generated and publicly available datasets are listed in the Supplementary Table S1.

## Code availability

All custom scripts used in this study are available at GitHub (https://github.com/jing-wan/Epromoter_GWAS) and Zenodo (https://zenodo.org/records/11061660) DOI 10.5281/zenodo.11061659.
