# Supplementary material for: Comprehensive mapping of genetic variation at Epromoters reveals pleiotropic association with multiple disease traits
Source: Nucleic Acids Res. 2024 Dec 27;53(4):gkae1270. doi: 10.1093/nar/gkae1270 (PMC11879118; doi:10.1093/nar/gkae1270)
Supplement: gkae1270_Supplemental_Files [file gkae1270_supplemental_files.zip › Supplemental Figures_V4.pptx]

## Slide 1
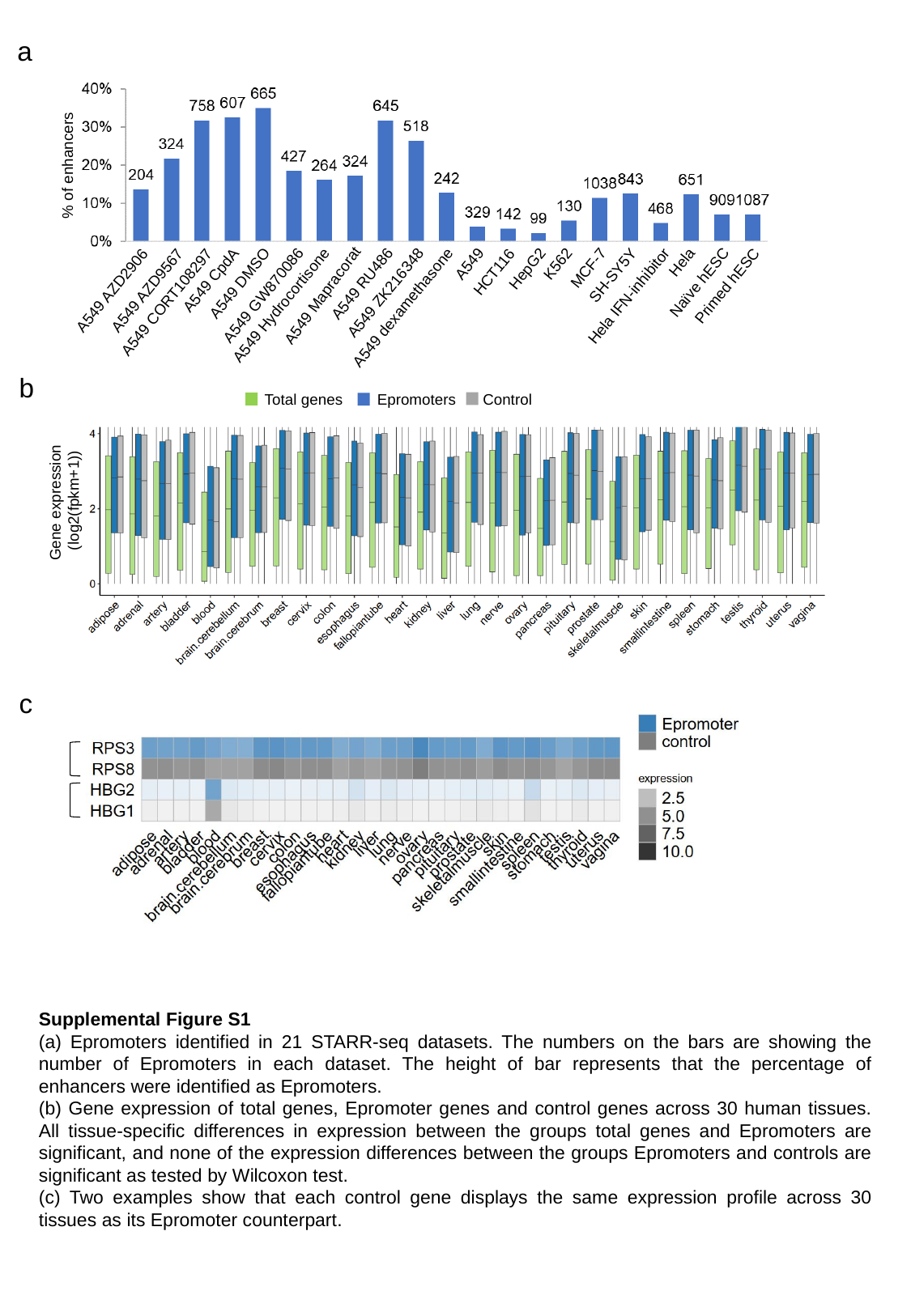

a
% of enhancers
Hela
A549
K562
MCF-7
HepG2
HCT116
SH-SY5Y
A549 CpdA
Naïve hESC
A549 DMSO
A549 RU486
Primed hESC
A549 AZD2906
A549 AZD9567
A549 ZK216348
A549 GW870086
A549 Mapracorat
Hela IFN-inhibitor
A549 CORT108297
A549 Hydrocortisone
A549 dexamethasone
b
Epromoters
Total genes
Control
Gene expression
(log2(fpkm+1))
c
Supplemental Figure S1
(a) Epromoters identified in 21 STARR-seq datasets. The numbers on the bars are showing the number of Epromoters in each dataset. The height of bar represents that the percentage of enhancers were identified as Epromoters.
(b) Gene expression of total genes, Epromoter genes and control genes across 30 human tissues. All tissue-specific differences in expression between the groups total genes and Epromoters are significant, and none of the expression differences between the groups Epromoters and controls are significant as tested by Wilcoxon test.
(c) Two examples show that each control gene displays the same expression profile across 30 tissues as its Epromoter counterpart.

## Slide 2
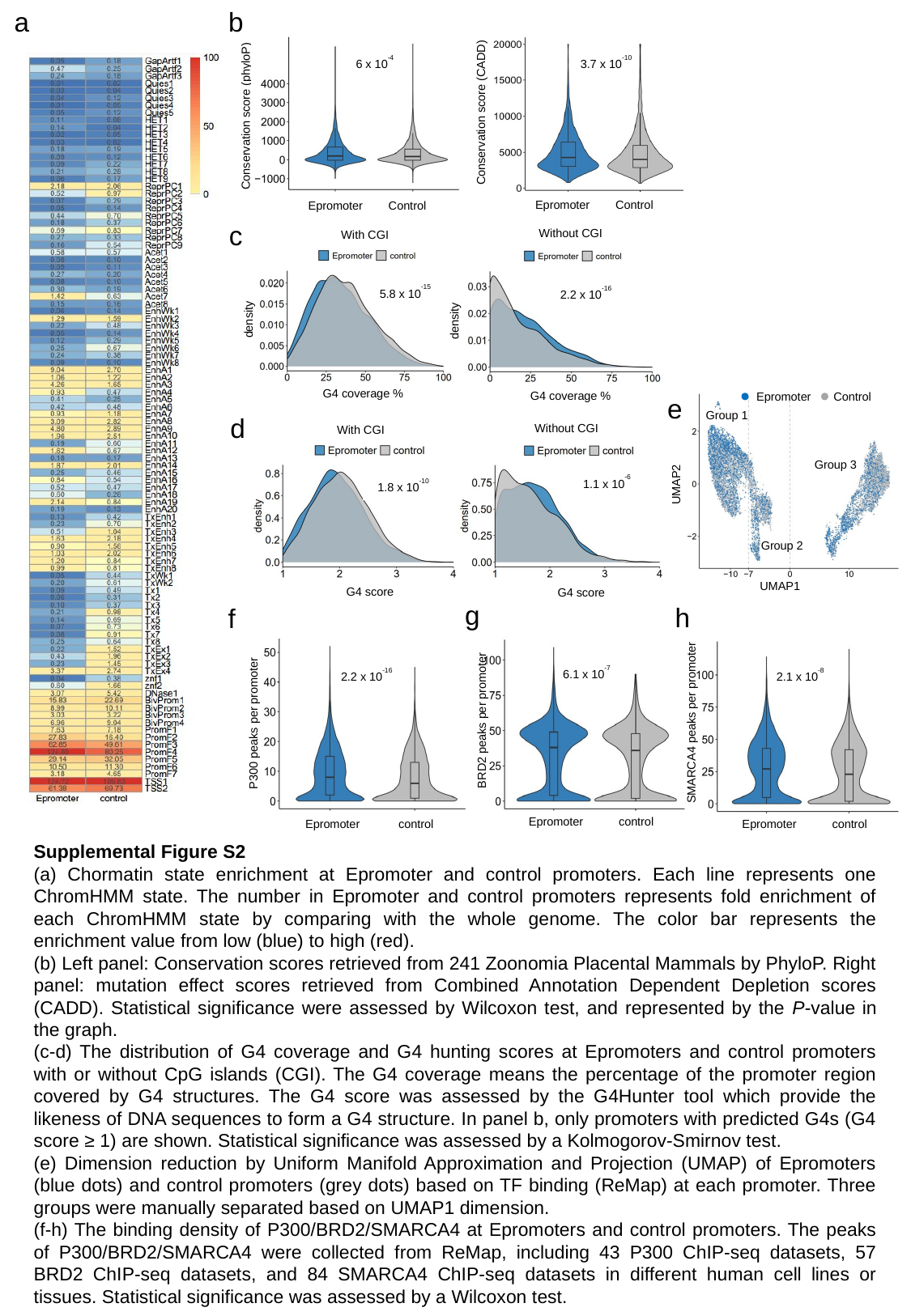

a
b
6 x 10^-4^
Conservation score (phyloP)
Epromoter Control
3.7 x 10^-10^
Conservation score (CADD)
Epromoter Control
c
Without CGI
With CGI
5.8 x 10^-15^
2.2 x 10^-16^
density
density
G4 coverage %
G4 coverage %
Epromoter Control
UMAP2
UMAP1
Group 1
Group 3
Group 2
e
d
Without CGI
With CGI
1.1 x 10^-6^
1.8 x 10^-10^
G4 score
G4 score
g
h
f
6.1 x 10^-7^
2.1 x 10^-8^
2.2 x 10^-16^
BRD2 peaks per promoter
SMARCA4 peaks per promoter
P300 peaks per promoter
Epromoter control
Epromoter control
Epromoter control
Supplemental Figure S2
(a) Chormatin state enrichment at Epromoter and control promoters. Each line represents one ChromHMM state. The number in Epromoter and control promoters represents fold enrichment of each ChromHMM state by comparing with the whole genome. The color bar represents the enrichment value from low (blue) to high (red).
(b) Left panel: Conservation scores retrieved from 241 Zoonomia Placental Mammals by PhyloP. Right panel: mutation effect scores retrieved from Combined Annotation Dependent Depletion scores (CADD). Statistical significance were assessed by Wilcoxon test, and represented by the P-value in the graph.
(c-d) The distribution of G4 coverage and G4 hunting scores at Epromoters and control promoters with or without CpG islands (CGI). The G4 coverage means the percentage of the promoter region covered by G4 structures. The G4 score was assessed by the G4Hunter tool which provide the likeness of DNA sequences to form a G4 structure. In panel b, only promoters with predicted G4s (G4 score ≥ 1) are shown. Statistical significance was assessed by a Kolmogorov-Smirnov test.
(e) Dimension reduction by Uniform Manifold Approximation and Projection (UMAP) of Epromoters (blue dots) and control promoters (grey dots) based on TF binding (ReMap) at each promoter. Three groups were manually separated based on UMAP1 dimension.
(f-h) The binding density of P300/BRD2/SMARCA4 at Epromoters and control promoters. The peaks of P300/BRD2/SMARCA4 were collected from ReMap, including 43 P300 ChIP-seq datasets, 57 BRD2 ChIP-seq datasets, and 84 SMARCA4 ChIP-seq datasets in different human cell lines or tissues. Statistical significance was assessed by a Wilcoxon test.

## Slide 3
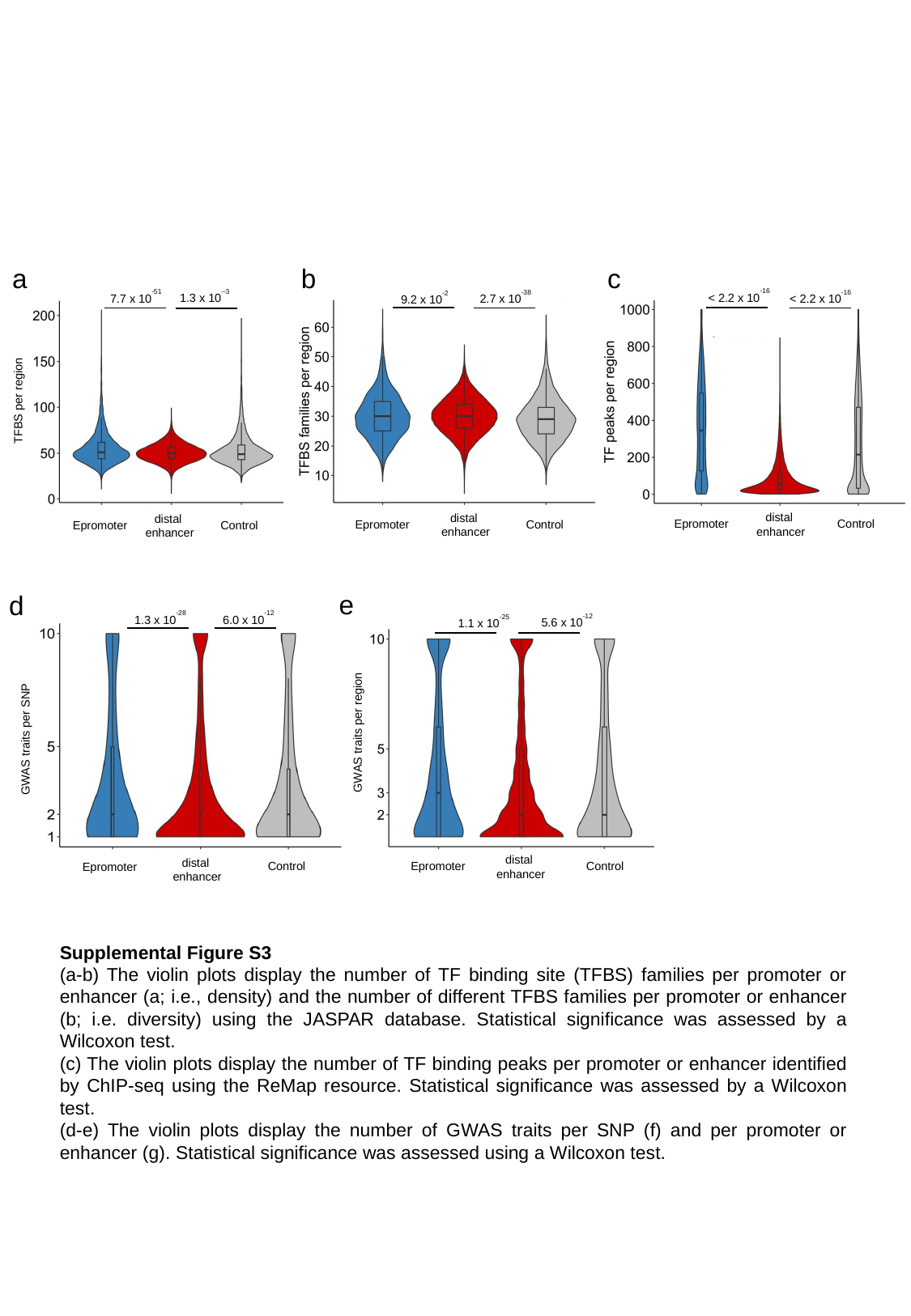

a
b
c
< 2.2 x 10^-16^
1.3 x 10--3
7.7 x 10^-51^
2.7 x 10^-38^
< 2.2 x 10^-16^
9.2 x 10^-2^
TFBS per region
distal
enhancer
Epromoter
Control
distal
enhancer
Epromoter
Control
distal
enhancer
Epromoter
Control
e
d
6.0 x 10^-12^
1.3 x 10^-28^
GWAS traits per SNP
distal
enhancer
Control
Epromoter
5.6 x 10^-12^
1.1 x 10^-25^
GWAS traits per region
distal
enhancer
Epromoter
Control
Supplemental Figure S3
(a-b) The violin plots display the number of TF binding site (TFBS) families per promoter or enhancer (a; i.e., density) and the number of different TFBS families per promoter or enhancer (b; i.e. diversity) using the JASPAR database. Statistical significance was assessed by a Wilcoxon test.
(c) The violin plots display the number of TF binding peaks per promoter or enhancer identified by ChIP-seq using the ReMap resource. Statistical significance was assessed by a Wilcoxon test.
(d-e) The violin plots display the number of GWAS traits per SNP (f) and per promoter or enhancer (g). Statistical significance was assessed using a Wilcoxon test.

## Slide 4
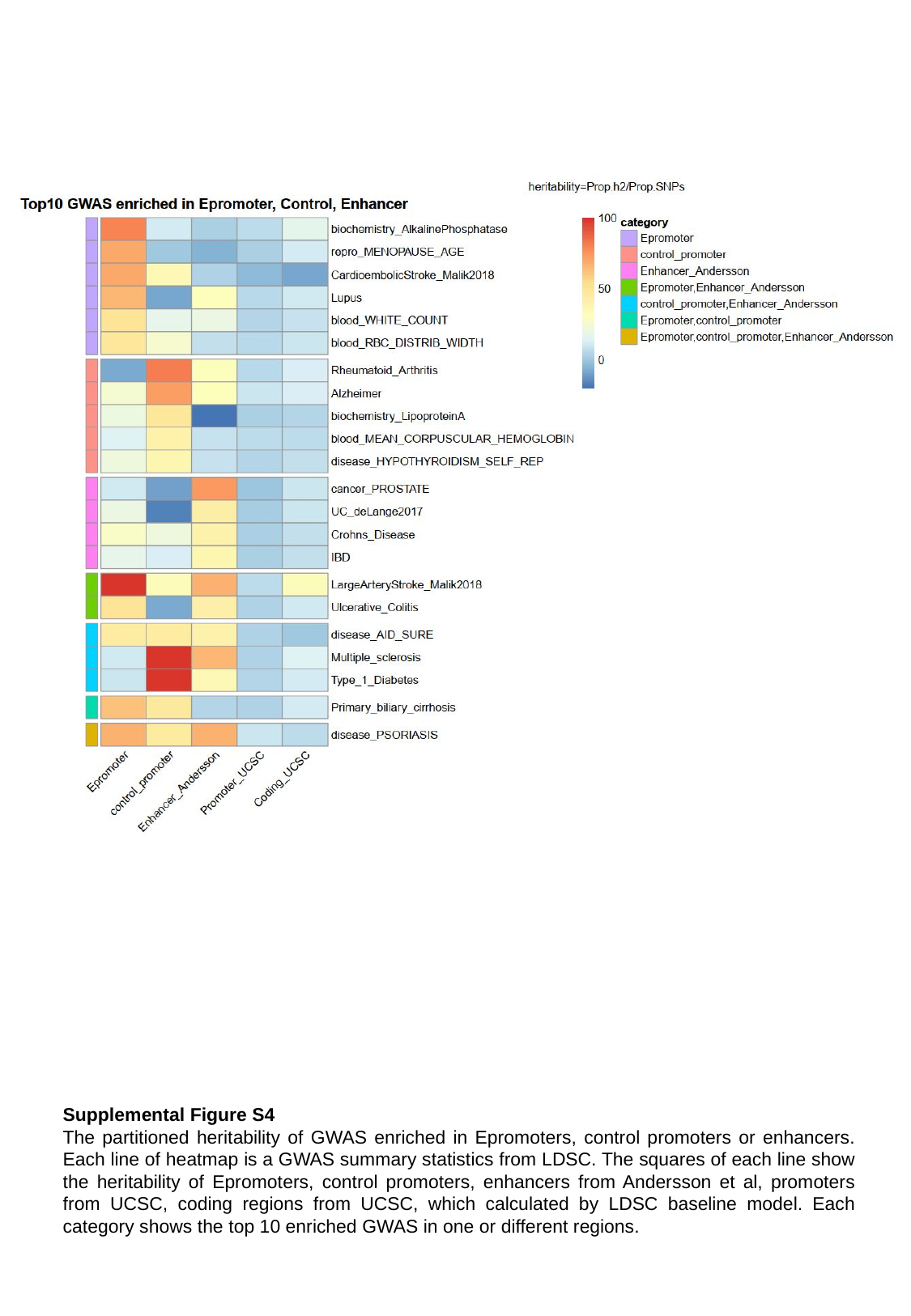

Supplemental Figure S4
The partitioned heritability of GWAS enriched in Epromoters, control promoters or enhancers. Each line of heatmap is a GWAS summary statistics from LDSC. The squares of each line show the heritability of Epromoters, control promoters, enhancers from Andersson et al, promoters from UCSC, coding regions from UCSC, which calculated by LDSC baseline model. Each category shows the top 10 enriched GWAS in one or different regions.

## Slide 5
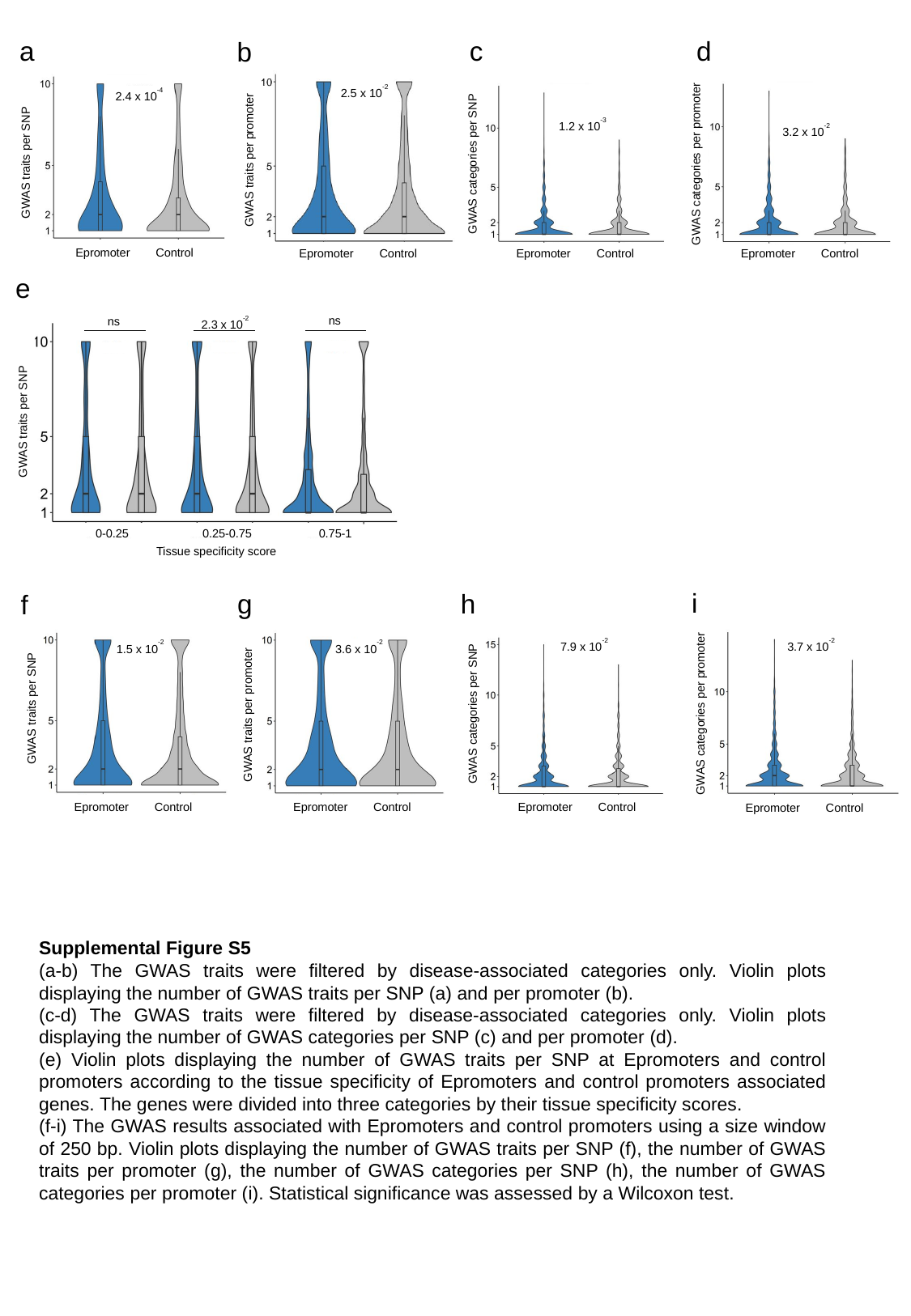

c
a
2.4 x 10^-4^
GWAS traits per SNP
Epromoter Control
d
b
GWAS traits per promoter
Epromoter Control
2.5 x 10^-2^
3.2 x 10^-2^
GWAS categories per promoter
Epromoter Control
1.2 x 10^-3^
GWAS categories per SNP
Epromoter Control
e
ns
2.3 x 10^-2^
ns
GWAS traits per SNP
0.75-1
0-0.25
0.25-0.75
Tissue specificity score
i
h
g
f
3.7 x 10^-2^
GWAS categories per promoter
Epromoter Control
1.5 x 10^-2^
GWAS traits per SNP
Epromoter Control
3.6 x 10^-2^
GWAS traits per promoter
Epromoter Control
7.9 x 10^-2^
GWAS categories per SNP
Epromoter Control
Supplemental Figure S5
(a-b) The GWAS traits were filtered by disease-associated categories only. Violin plots displaying the number of GWAS traits per SNP (a) and per promoter (b).
(c-d) The GWAS traits were filtered by disease-associated categories only. Violin plots displaying the number of GWAS categories per SNP (c) and per promoter (d).
(e) Violin plots displaying the number of GWAS traits per SNP at Epromoters and control promoters according to the tissue specificity of Epromoters and control promoters associated genes. The genes were divided into three categories by their tissue specificity scores.
(f-i) The GWAS results associated with Epromoters and control promoters using a size window of 250 bp. Violin plots displaying the number of GWAS traits per SNP (f), the number of GWAS traits per promoter (g), the number of GWAS categories per SNP (h), the number of GWAS categories per promoter (i). Statistical significance was assessed by a Wilcoxon test.

## Slide 6
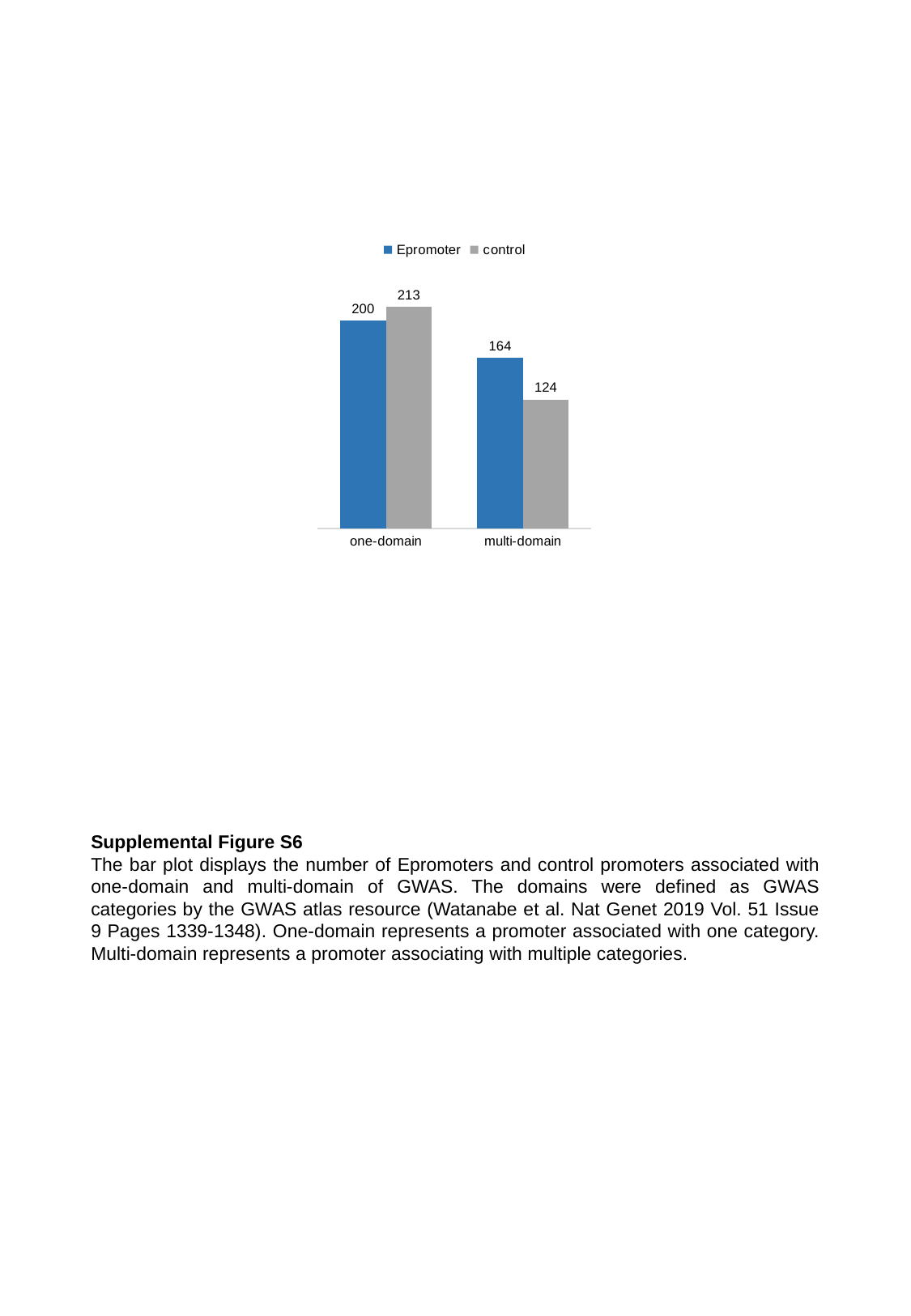

### Chart
| Category | Epromoter | control |
|---|---|---|
| one-domain | 200.0 | 213.0 |
| multi-domain | 164.0 | 124.0 |Supplemental Figure S6
The bar plot displays the number of Epromoters and control promoters associated with one-domain and multi-domain of GWAS. The domains were defined as GWAS categories by the GWAS atlas resource (Watanabe et al. Nat Genet 2019 Vol. 51 Issue 9 Pages 1339-1348). One-domain represents a promoter associated with one category. Multi-domain represents a promoter associating with multiple categories.

## Slide 7
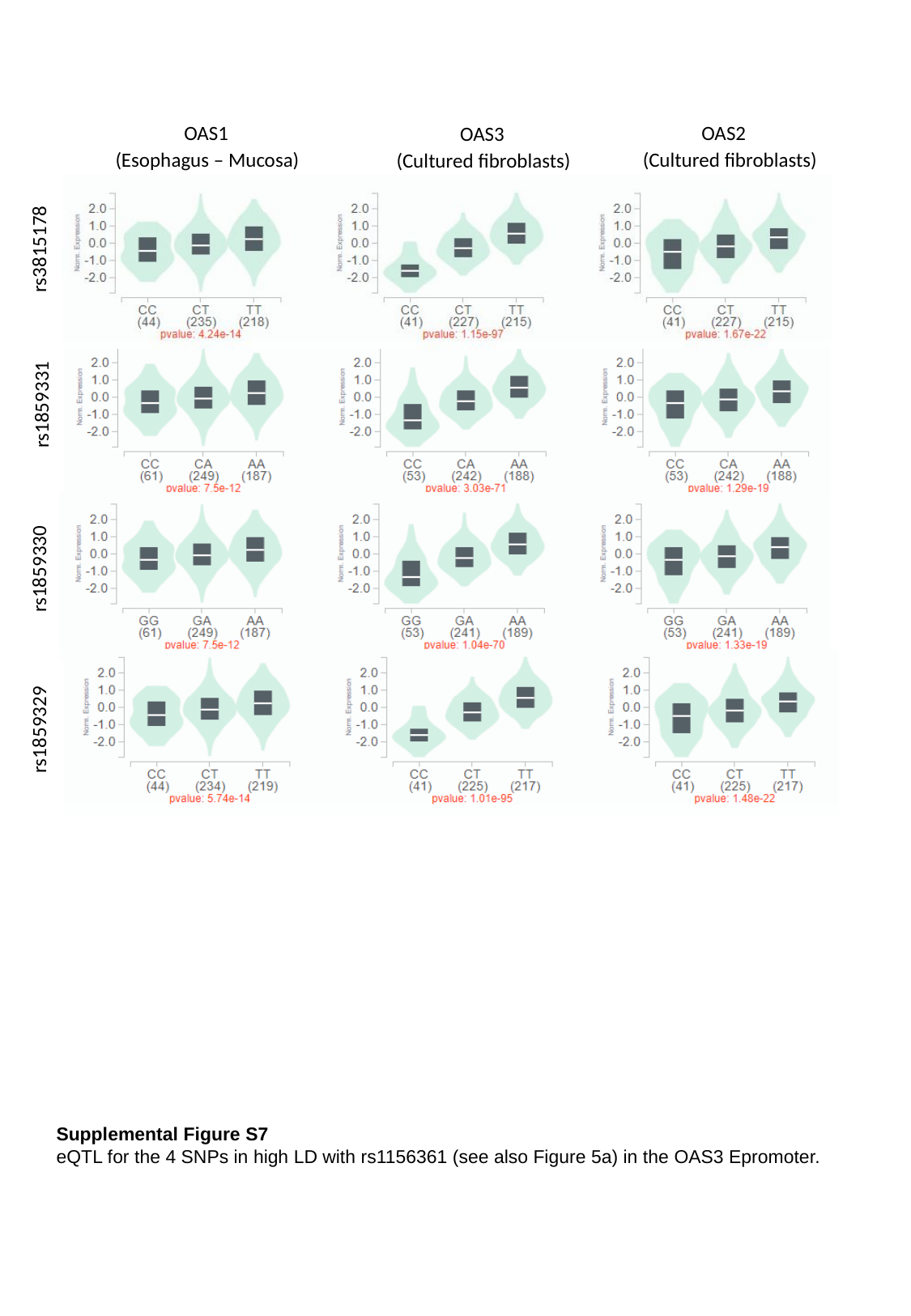

OAS1
OAS2
OAS3
(Esophagus – Mucosa)
(Cultured fibroblasts)
(Cultured fibroblasts)
rs3815178
rs1859331
rs1859330
rs1859329
Supplemental Figure S7
eQTL for the 4 SNPs in high LD with rs1156361 (see also Figure 5a) in the OAS3 Epromoter.
