# Supplementary material for: Comprehensive mapping of genetic variation at Epromoters reveals pleiotropic association with multiple disease traits
Source: Nucleic Acids Res. 2024 Dec 27;53(4):gkae1270. doi: 10.1093/nar/gkae1270 (PMC11879118; doi:10.1093/nar/gkae1270)
Supplement: gkae1270_Supplemental_Files [file gkae1270_supplemental_files.zip › Supplemental information 1_v6.docx]

**Supplemental information 1:** Physiological and pathological relevancies of four Epromoter loci

***SETD1A* locus**

The *SETD1A* Epromoter is active in A549 upon 4 different conditions (DMSO, CORT108297, CpdA and RU486), as well as in naïve hESCs.

Rs4889599 lies in this Epromoter, and is in LD with SNPs that are associated with 30 different GWAS (Graves’ disease, Juvenile myoclonic epilepsy, Parkinson's disease, ankylosing spondylitis, psoriasis, ulcerative colitis, Crohn's disease, sclerosing cholangitis, aspartate aminotransferase measurement, serum alanine aminotransferase measurement, low density lipoprotein triglyceride measurement, body fat percentage, high density lipoprotein cholesterol measurement, sex hormone-binding globulin measurement, body fat distribution, body fat percentage, body height, body mass index, diastolic blood pressure, eosinophil count, erythrocyte count, gait measurement, heel bone mineral density, hematocrit, hip circumference, irritability measurement, lifestyle measurement, mean corpuscular hemoglobin, mean corpuscular volume, mean reticulocyte volume, multiple sclerosis, neuroticism measurement, psoriasis, psoriatic arthritis, pulse pressure measurement, response to anticoagulant, sex hormone-binding globulin measurement, total blood protein measurement, triglyceride measurement, visceral adipose tissue measurement, vitamin D measurement, waist circumference, tonsillectomy risk measurement), including immune-associated diseases (Graves, psoriasis, Crohn’s, eosinophil count), neurologic diseases (Parkinson’s, epilepsy, anxiety) and heart disease risk factors (BMI, blood and pulse pressure, triglycerides and LDL cholesterol measurements).

There are 30 P-P-interactions in different tissues (*AC135050.5*, *ZNF668*, *ARMC5*, *COX6A2*, *FUS*, *RP11-388M20.6*, *ITGAD*, *KAT8*, *RP11-196G11.4*, *PRSS36*, *PRSS53*, *RP11-196G11.1*, *VKORC1*, *RP11-170L3.2*, *ZNF267*, *ZNF646, ZNF668*, *ARMC5*, *COX6A2, FUS*, *ITGAD*, *ITGAX*, *PRSS36*, *RNF40*, *SLC5A2*, *STX1B*, *TGFB1I1*, *ZNF267*, *ZNF629*, *ZNF646*, *ZNF668*, *ZNF771*, *ZNF843*, *ORAI3*, *SETD1A*), and 6 genes in eQTL with the SNP (*HSD3B7*, *STX1B*, *STX4*, *PRSS53*, *VKORC1* and *KAT8*) (Figure 7a-b). The eQTLs show that the alternative allele decreases expression of all of the eQTLs except *VKORC1*, which shows increased expression. However, *SETD1A* is not an eQTL. Moreover, MPRA from the Mattioli et col. study (1) found consistent decreased activity of the alternative allele of rs4889599 in K562 and HepG2 cell lines (Figure 7c). Interestingly, the variant changes TFBS for several TFs, including HTATIP2 (Figure 7d), which is a positive regulator of transcription by RNA polymerase II, and EGR1 and EGR2 (Supplemental Table 5), transcriptional regulators that are involved in ischemia response and repression of inflammatory enhancers (2), which could contribute to the immune-related disease associations.

The *SETD1A* gene encodes a chromatin modifier protein involved in synaptic function and development of neurons, and mutations in this gene are associated with early-onset epilepsy (3), which makes this gene also a candidate for contribution to other neurodevelopmental disorder associations like Parkinson’s and anxiety.

HSD3B7 is an enzyme involved in the synthesis of bile acids from cholesterol, but also plays a role in lymphoid cell movement by regulating a chemotactic receptor (4). As such this eQTL target could be involved in any of the immune related associations.

STX1B Syntaxin 1B plays a role in exocytosis and synaptic vesicles, and has been associated with a spectrum of epilepsy syndromes (5), as well as Parkinson’s disease (6).

*STX4* Syntaxin 4 is a paralog of *STX1B*, is also involved in synaptic vesicle biology, and is associated with a range of cardiometabolic pathologies, including triglyceride level systolic blood pressure and body fat percentage (7-9), which could also be the link with the cardiometabolic GWAS linked to rs4889599 and Epromoter *SETD1A*. Additionally, *STX4* is expressed in pancreatic B cells, promotes islet function (10) and could thus play a role in the diabetes association . Moreover, *STX4* is involved in cytotoxic T-lymphocyte immunological synapse formation (11), which could mediate the auto-immune associations (e.g. Graves’, Crohn’s, psoriasis, but also neurodegenerative diseases like Parkinson’s).

PRSS53 is predicted to have endopeptidase activity and was identified to play a role in maintaining the health of pancreatic islet b cells (12). This gene is associated with psoriasis, and it is the most highly over-expressed gene in psoriatic skin (13), potentially mediated through the allele-specific *SETD1A* Epromoter.

Overexpression of *VKORC1* was identified to lead to increased VKOR activity, which is the target of anticoagulants, thus leading to its association “response to anticoagulant drugs” that could be mediated by the SNP in Epromoter *SETD1A* (14).

***ORMDL3* locus**

The ***ORMDL3*** Epromoter in active in naïve hESCs, and SNPs rs4065275 and rs8076131 in this region are in LD with SNPs associated with over 30 GWAS hits (Crohn's disease, Eczema, allergic rhinitis, Glucocorticoid use measurement, Inhalant adrenergic use measurement, Oral ulcer, acute lymphoblastic leukemia, age at onset of asthma, allergic rhinitis, allergy, allergy age at onset, asthma, ankylosing spondylitis, psoriasis, ulcerative colitis, sclerosing cholangitis, asthma, asthma exacerbation measurement, atopic asthma, atrial fibrillation, autoimmune disease, autoimmune thyroid disease, type I diabetes mellitus, Common variable immunodeficiency, ankylosing spondylitis, psoriasis, celiac disease, ulcerative colitis, juvenile idiopathic arthritis, systemic lupus erythematosus, biliary liver cirrhosis, bipolar I disorder, blood protein measurement, cervical carcinoma, childhood onset asthma, atopic eczema, atopic march, dermatomyositis, juvenile dermatomyositis, eosinophil count, eosinophil percentage of leukocytes, inflammatory bowel disease, leukocyte count, lymphocyte count, mathematical ability, monocyte percentage of leukocytes, multiple sclerosis, nitric oxide exhalation measurement, platelet-to-lymphocyte ratio, primary biliary cirrhosis, respiratory system disease, rheumatoid arthritis, selective IgA deficiency disease, self-reported educational attainment, serum IgM measurement, serum gamma-glutamyl transferase measurement, serum non-albumin protein measurement, systemic scleroderma, ulcerative colitis), the majority of which are a wide range of auto-immune related diseases (e.g. asthma, eczema, allergy, Crohn’s disease, type I diabetes, SLE, RA).

eQTLs of rs4065275 are *AC090844.2*, *GSDMA*, *GSDMB*, *IKZF3*, *ORMDL3*. eQTLs of rs8076131 are *PGAP3*, *IKZF3*, *GSDMB*, *GSDMA* and *ORDML3*. P-P interactions are found with 8 genes (*AC087491.2*, *PPP1R1B*, *ERBB2*, *PGAP3*, *GRB7*, *IKZF3*, *ZPBP2*, *MIEN1*), which include common targets with eQTL *IKZF3* and *PGAP3* (Figure 7a-b). CRISPRi data has shown that *KRT10* and its antisense-RNA (TMEM99) are regulatory targets of the *ORMDL3* promoter. KRT10 (keratin) is a component of the cytoskeleton of skin epithelial cells, and it plays a role in microbial infection in the nose and lung (15). The alternative allele of rs4065275 was found to increase regulatory activity in an MPRA in Jurkat cells (Figure 7c) (16), while the alternative allele of rs8076131 showed a decrease in regulatory activity in an MPRA in HEK293T cells (17). A link with the GWAS could be the fact that the alternative variant of rs4065275 changes TFBS for IKZF2, a member of the IKAROS transcription factor family which is involved in the regulation of lymphocyte development and controls T cell apoptosis in an IL2-dependent manner (Figure 7d) (18,19).

*ORDML3* plays a role in innate immunity, explaining its involvement in most of the auto-immune related disease-associations. Additionally, increased expression of *ORMDL3* has been associated with asthma (20). The alternative allele of the variants is associated with increased expression of *ORMDL3* as well as *GSDMA* and *GSDMB* in several tissues (eQTL), but interestingly with decreased expression of *IKZF3*, another member of the IKAROS transcription factor family. Indeed, altered expression of this transcription factor due to the variants in the *ORDML3* Epromoter could explain the wide range of immune-related diseases associated with the variants.

*PGAP3* encodes a glycosylphosphatidylinositol-specific phospholipase. Mutations in this gene cause neurologic hyperphosphatasia with cognitive disability (21), and might be linked with cognitive-associated GWAS like bipolar I disorder, mathematical ability and educational attainment via a similar biological mechanism.

*GSDMA* (Gasdermin A) and *GSDMB* (Gasdermin B) are involved in inflammatory cell death (pyroptosis), necessary for the recruitment of immune cells to infected sites in the skin and intestine (22,23). Altered expression of these genes as indicated by eQTL for both variants could be linked to eczema and intestine-related autoimmune disease association.

***COASY* locus**

The *COASY* promoter shows enhancer activity in 5 different cell lines (A549, CCRF CEM with IFNα stimulation, HCT116, MCF-7, and SH-SY5Y). SNP rs629861 in the *COASY* promoter is associated with 17 different GWAS traits (Drugs used in diabetes use measurement, Eczema, Parkinson's disease-age at diagnosis, type II diabetes mellitus, atopic asthma, body mass index, body weight, colorectal cancer, endometrial neoplasm, cortical surface area measurement, high density lipoprotein cholesterol measurement, lymphocyte count, self-reported educational attainment, serum gamma-glutamyl transferase measurement, tea consumption measurement, vitamin D measurement). Disease ontology includes Parkinson’s disease, asthma, type II diabetes and body mass index.

SNP rs629861 has 7 eQTLs within 100kb (*CNTNAP1, COASY, HSD17B1, PLEKHH3, PSMC3IP, TUBG1, TUBG2*), and the COASY promoter has 22 P-P interactions in over 30 tissues (*AC003104.1, ATP6V0A1, CCR10, CNTNAP1, CTD-3193K9.4, PLEKHH3, CNP, CNTD1, COA3, CTD-2132N18.3, RAB5C, DNAJC7, NKIRAS2, FAM134C, TUBG1, HMGN2P15, PTRF, TUBG2, WNK4, COASY, LOC108783654, MLX*). As many as 5 gene targets overlap between the eQTL and P-P interactions (*CNTNAP1, COASY, PLEKHH3, TUBG1, TUBG2*) (Figure 7a-b), increasing the likelihood that these genes are potential targets of the *COASY* Epromoter. The SNP shows allelic-skewed activity in K562 cells as assessed by MPRA (24) (Figure 7c). The SNP alters the binding sites of several TFBS, including loss of the GLTPD1 binding site (Figure 7d), which is involved in negative regulation of NLRP3 inflammasome complex assembly and interleukin-1 beta production. Moreover, the SNP also alters the binding of HCFC1 (Supplemental Table 5), which is required for certain types of insulin secretion (25), as well as for the recruitment of epigenetic activators to promoters of lipogenic genes to promote *de novo* lipogenesis (26). These functions of HCFC1 could link the SNP to the metabolic-related GWAS type II diabetes, body weight and body mass index.

*COASY* encodes protein coenzyme A synthase, which plays an important role in synthetic and degradative metabolic pathways, in particular of vitamin B5 (27). Mutations in this gene are associated with neurodegeneration with brain iron accumulation (28-30), which could imply similar mechanisms are at play in explaining the association of rs629861 with Parkinson’s disease. Furthermore, contactin-associated protein (*CNTNAP1*) is also associated with hypomyelination and nervous system development (31), and could therefore as well be a distal target of the *COASY* Epromoter explaining the association with Parkinson’s disease. *HSD17B1* and *PSMC3IP*, both eQTLs for rs629861, are involved in estrogen metabolism and activation (32,33). Because estrogen is a known player in insulin sensitivity and gluconeogenesis (34), this gene could be a distal target of the *COASY* Epromoter involved in the metabolic- and sex-hormone related traits (endometrial neoplasm, body weight, lipoprotein cholesterol, and type II diabetes).

***NIF3L1/PPIL3* locus**

The shared *NIF3L1/PPIL3* promoter shows enhancer activity in 2 different cell types (SH-SY5Y_normal and primed hESC). A variant in this Epromoter is rs7559150, which is associated with 3 different GWAS (outer ear morphology trait, parathyroid hormone measurement, response to triptolide). Triptolide is a compound from the bark of a plant root that has anti-inflammatory properties and it has been used in the treatment of autoimmune diseases, fibrosis and neurodegeneration.

There are 3 genes in eQTL with rs7559150 (*NIF3L1*, *PPIL3* and *CFLAR*). Additionally, 21 genes show P-P interaction with the Epromoter in different tissues (*ALS2, ALS2CR12, CASP10, CASP8, CFLAR, CFLAR-AS1, FAM126B, NDUFB3, RNU6-1206P, KCTD18, SGOL2, NOP58, SNORD70, STRADB, TRAK2, BZW1, CLK1, FAM126B, LOC101927795, NIF3L1, PPIL3*) (Figure 7a-b). Transcription of two genes is impacted by CRISPRi on the Epromoter (*PPIL3* and *CFLAR*)*,* giving additional evidence for Epromoter activity and target genes. The minor allele of the SNP is associated with a significant decrease in the expression of *PPIL3, NIF3L1* and *CFLAR*, as well as several other genes in the vicinity. *CFLAR* is an eQTL of the SNP with an increase in expression in the esophagus, but a decrease in expression in e.g. thyroid. Similarly, the minor allele of rs7559150 showed decreased regulatory activity in a SuRE assay in K562 cells (Figure 7c)(24). Several TFBS are disrupted by rs7559150, including several members of the ETS family of TFs (Figure 7d; Supplemental Table 5), which are involved in a wide range of functions including inflammation and apoptosis, which could be at the basis of the association with triptolide cytotoxicity.

The rs7559150 SNP was identified to reduce *NIF3L1* expression in memory T cells after 16h of stimulation (35). Moreover, the gene *NIF3L1* itself is associated with Williams-Beuren syndrome, in which patients often have elevated blood calcium levels (hypercalcemia) (36). This is in agreement with the fact that rs7559150 is associated with parathyroid hormone measurement. The rs7559150 minor allele could lead to decreased *NIF3L1* expression which in turn leads to decreased negative regulation of transcription of the target genes, resulting in increased parathyroid activity and increased blood calcium levels. Additionally, *NIF3L1* lies in the ALS2-critical region, a neurodegenerative disease with loss of motor neurons. Interestingly, one of the P-P interactions of this Epromoter is with the *ALS2* gene, mutations in which are also associated with ALS. Potentially the *NIF3L1/PPIL3* Epromoter could play a role in the regulation of *ALS2* (over 600kb away), adding to the ALS-association of both genes.

*CFLAR* is a gene regulator of apoptosis and inflammation (37,38). Moreover, high expression of *CFLAR* was found to positively regulate immune response to soft tissue sarcoma in the tumor microenvironment (39). Rs7559150 in Epromoter *NIF3L1/PPIL3* shows P-P interaction with *CFLAR*, and could thus potentially regulate and alter expression of *CFLAR*, resulting in an altered systemic apoptosis and inflammation regulation, which could culminate in altered cytotoxicity and its association with triptolide response.

The PPIL3 protein is a member of the cyclophilin family, and was shown to be a negative feedback regulator of NF-kB signaling pathway in homeostasis of innate immunity (40), which could similarly suggest involvement in the association to triptolide cytotoxicity.

**References**

1. Mattioli, K., Volders, P.J., Gerhardinger, C., Lee, J.C., Maass, P.G., Mele, M. and Rinn, J.L. (2019) High-throughput functional analysis of lncRNA core promoters elucidates rules governing tissue specificity. *Genome Res*, **29**, 344-355.

2. Trizzino, M., Zucco, A., Deliard, S., Wang, F., Barbieri, E., Veglia, F., Gabrilovich, D. and Gardini, A. (2021) EGR1 is a gatekeeper of inflammatory enhancers in human macrophages. *Sci Adv*, **7**.

3. Yu, X., Yang, L., Li, J., Li, W., Li, D., Wang, R., Wu, K., Chen, W., Zhang, Y., Qiu, Z. and Zhou, W. (2019) De Novo and Inherited SETD1A Variants in Early-onset Epilepsy. *Neurosci Bull*, **35**, 1045-1057.

4. Yi, T., Wang, X., Kelly, L.M., An, J., Xu, Y., Sailer, A.W., Gustafsson, J.A., Russell, D.W. and Cyster, J.G. (2012) Oxysterol gradient generation by lymphoid stromal cells guides activated B cell movement during humoral responses. *Immunity*, **37**, 535-548.

5. Schubert, J., Siekierska, A., Langlois, M., May, P., Huneau, C., Becker, F., Muhle, H., Suls, A., Lemke, J.R., de Kovel, C.G. *et al.* (2014) Mutations in STX1B, encoding a presynaptic protein, cause fever-associated epilepsy syndromes. *Nat Genet*, **46**, 1327-1332.

6. Nalls, M.A., Pankratz, N., Lill, C.M., Do, C.B., Hernandez, D.G., Saad, M., DeStefano, A.L., Kara, E., Bras, J., Sharma, M. *et al.* (2014) Large-scale meta-analysis of genome-wide association data identifies six new risk loci for Parkinson's disease. *Nat Genet*, **46**, 989-993.

7. Richardson, T.G., Sanderson, E., Palmer, T.M., Ala-Korpela, M., Ference, B.A., Davey Smith, G. and Holmes, M.V. (2020) Evaluating the relationship between circulating lipoprotein lipids and apolipoproteins with risk of coronary heart disease: A multivariable Mendelian randomisation analysis. *PLoS Med*, **17**, e1003062.

8. Sakaue, S., Kanai, M., Tanigawa, Y., Karjalainen, J., Kurki, M., Koshiba, S., Narita, A., Konuma, T., Yamamoto, K., Akiyama, M. *et al.* (2021) A cross-population atlas of genetic associations for 220 human phenotypes. *Nat Genet*, **53**, 1415-1424.

9. Martin, S., Cule, M., Basty, N., Tyrrell, J., Beaumont, R.N., Wood, A.R., Frayling, T.M., Sorokin, E., Whitcher, B., Liu, Y. *et al.* (2021) Genetic Evidence for Different Adiposity Phenotypes and Their Opposing Influences on Ectopic Fat and Risk of Cardiometabolic Disease. *Diabetes*, **70**, 1843-1856.

10. Oh, E., Ahn, M., Afelik, S., Becker, T.C., Roep, B.O. and Thurmond, D.C. (2018) Syntaxin 4 Expression in Pancreatic β-Cells Promotes Islet Function and Protects Functional β-Cell Mass. *Diabetes*, **67**, 2626-2639.

11. Spessott, W.A., Sanmillan, M.L., Kulkarni, V.V., McCormick, M.E. and Giraudo, C.G. (2017) Syntaxin 4 mediates endosome recycling for lytic granule exocytosis in cytotoxic T-lymphocytes. *Traffic*, **18**, 442-452.

12. Mizusawa, N., Harada, N., Iwata, T., Ohigashi, I., Itakura, M. and Yoshimoto, K. (2022) Identification of protease serine S1 family member 53 as a mitochondrial protein in murine islet beta cells. *Islets*, **14**, 1-13.

13. Stuart, P.E., Nair, R.P., Ellinghaus, E., Ding, J., Tejasvi, T., Gudjonsson, J.E., Li, Y., Weidinger, S., Eberlein, B., Gieger, C. *et al.* (2010) Genome-wide association analysis identifies three psoriasis susceptibility loci. *Nat Genet*, **42**, 1000-1004.

14. Rost, S., Fregin, A., Ivaskevicius, V., Conzelmann, E., Hörtnagel, K., Pelz, H.J., Lappegard, K., Seifried, E., Scharrer, I., Tuddenham, E.G. *et al.* (2004) Mutations in VKORC1 cause warfarin resistance and multiple coagulation factor deficiency type 2. *Nature*, **427**, 537-541.

15. Shivshankar, P., Boyd, A.R., Le Saux, C.J., Yeh, I.T. and Orihuela, C.J. (2011) Cellular senescence increases expression of bacterial ligands in the lungs and is positively correlated with increased susceptibility to pneumococcal pneumonia. *Aging Cell*, **10**, 798-806.

16. Mouri, K., Guo, M.H., de Boer, C.G., Lissner, M.M., Harten, I.A., Newby, G.A., DeBerg, H.A., Platt, W.F., Gentili, M., Liu, D.R. *et al.* (2022) Prioritization of autoimmune disease-associated genetic variants that perturb regulatory element activity in T cells. *Nat Genet*, **54**, 603-612.

17. Liu, S., Liu, Y., Zhang, Q., Wu, J., Liang, J., Yu, S., Wei, G.H., White, K.P. and Wang, X. (2017) Systematic identification of regulatory variants associated with cancer risk. *Genome Biol*, **18**, 194.

18. Morgan, B., Sun, L., Avitahl, N., Andrikopoulos, K., Ikeda, T., Gonzales, E., Wu, P., Neben, S. and Georgopoulos, K. (1997) Aiolos, a lymphoid restricted transcription factor that interacts with Ikaros to regulate lymphocyte differentiation. *EMBO J*, **16**, 2004-2013.

19. Heizmann, B., Kastner, P. and Chan, S. (2018) The Ikaros family in lymphocyte development. *Curr Opin Immunol*, **51**, 14-23.

20. Nowakowska, J., Olechnowicz, A., Langwiński, W., Koteluk, O., Lemańska, Ż., Jóźwiak, K., Kamiński, K., Łosiewski, W., Stegmayr, J., Wagner, D. *et al.* (2023) Increased expression of ORMDL3 in allergic asthma: a case control and in vitro study. *J Asthma*, **60**, 458-467.

21. Abdel-Hamid, M.S., Issa, M.Y., Otaify, G.A., Abdel-Ghafar, S.F., Elbendary, H.M. and Zaki, M.S. (2018) PGAP3-related hyperphosphatasia with mental retardation syndrome: Report of 10 new patients and a homozygous founder mutation. *Clin Genet*, **93**, 84-91.

22. Deng, W., Bai, Y., Deng, F., Pan, Y., Mei, S., Zheng, Z., Min, R., Wu, Z., Li, W., Miao, R. *et al.* (2022) Streptococcal pyrogenic exotoxin B cleaves GSDMA and triggers pyroptosis. *Nature*, **602**, 496-502.

23. Zhou, Z., He, H., Wang, K., Shi, X., Wang, Y., Su, Y., Li, D., Liu, W., Zhang, Y., Shen, L. *et al.* (2020) Granzyme A from cytotoxic lymphocytes cleaves GSDMB to trigger pyroptosis in target cells. *Science*, **368**.

24. van Arensbergen, J., Pagie, L., FitzPatrick, V.D., de Haas, M., Baltissen, M.P., Comoglio, F., van der Weide, R.H., Teunissen, H., Vosa, U., Franke, L. *et al.* (2019) High-throughput identification of human SNPs affecting regulatory element activity. *Nat Genet*, **51**, 1160-1169.

25. Iwata, T.N., Cowley, T.J., Sloma, M., Ji, Y., Kim, H., Qi, L. and Lee, S.S. (2013) The transcriptional co-regulator HCF-1 is required for INS-1 beta-cell glucose-stimulated insulin secretion. *PLoS One*, **8**, e78841.

26. Lane, E.A., Choi, D.W., Garcia-Haro, L., Levine, Z.G., Tedoldi, M., Walker, S. and Danial, N.N. (2019) HCF-1 Regulates De Novo Lipogenesis through a Nutrient-Sensitive Complex with ChREBP. *Mol Cell*, **75**, 357-371 e357.

27. Daugherty, M., Polanuyer, B., Farrell, M., Scholle, M., Lykidis, A., de Crécy-Lagard, V. and Osterman, A. (2002) Complete reconstitution of the human coenzyme A biosynthetic pathway via comparative genomics. *J Biol Chem*, **277**, 21431-21439.

28. Dusi, S., Valletta, L., Haack, T.B., Tsuchiya, Y., Venco, P., Pasqualato, S., Goffrini, P., Tigano, M., Demchenko, N., Wieland, T. *et al.* (2014) Exome sequence reveals mutations in CoA synthase as a cause of neurodegeneration with brain iron accumulation. *Am J Hum Genet*, **94**, 11-22.

29. van Dijk, T., Ferdinandusse, S., Ruiter, J.P.N., Alders, M., Mathijssen, I.B., Parboosingh, J.S., Innes, A.M., Meijers-Heijboer, H., Poll-The, B.T., Bernier, F.P. *et al.* (2018) Biallelic loss of function variants in COASY cause prenatal onset pontocerebellar hypoplasia, microcephaly, and arthrogryposis. *Eur J Hum Genet*, **26**, 1752-1758.

30. Rosati, J., Johnson, J., Stander, Z., White, A., Tortorelli, S., Bailey, D., Fong, C.T. and Lee, B.H. (2023) Progressive brain atrophy and severe neurodevelopmental phenotype in siblings with biallelic COASY variants. *Am J Med Genet A*, **191**, 842-845.

31. Laquérriere, A., Maluenda, J., Camus, A., Fontenas, L., Dieterich, K., Nolent, F., Zhou, J., Monnier, N., Latour, P., Gentil, D. *et al.* (2014) Mutations in CNTNAP1 and ADCY6 are responsible for severe arthrogryposis multiplex congenita with axoglial defects. *Hum Mol Genet*, **23**, 2279-2289.

32. Puranen, T., Poutanen, M., Ghosh, D., Vihko, P. and Vihko, R. (1997) Characterization of structural and functional properties of human 17 beta-hydroxysteroid dehydrogenase type 1 using recombinant enzymes and site-directed mutagenesis. *Molecular endocrinology (Baltimore, Md.)*, **11**, 77-86.

33. Zangen, D., Kaufman, Y., Zeligson, S., Perlberg, S., Fridman, H., Kanaan, M., Abdulhadi-Atwan, M., Abu Libdeh, A., Gussow, A., Kisslov, I. *et al.* (2011) XX ovarian dysgenesis is caused by a PSMC3IP/HOP2 mutation that abolishes coactivation of estrogen-driven transcription. *Am J Hum Genet*, **89**, 572-579.

34. Yan, H., Yang, W., Zhou, F., Li, X., Pan, Q., Shen, Z., Han, G., Newell-Fugate, A., Tian, Y., Majeti, R. *et al.* (2019) Estrogen Improves Insulin Sensitivity and Suppresses Gluconeogenesis via the Transcription Factor Foxo1. *Diabetes*, **68**, 291-304.

35. Soskic, B., Cano-Gamez, E., Smyth, D.J., Ambridge, K., Ke, Z., Matte, J.C., Bossini-Castillo, L., Kaplanis, J., Ramirez-Navarro, L., Lorenc, A. *et al.* (2022) Immune disease risk variants regulate gene expression dynamics during CD4. *Nat Genet*, **54**, 817-826.

36. Merla, G., Howald, C., Antonarakis, S.E. and Reymond, A. (2004) The subcellular localization of the ChoRE-binding protein, encoded by the Williams-Beuren syndrome critical region gene 14, is regulated by 14-3-3. *Hum Mol Genet*, **13**, 1505-1514.

37. Xiaohong, W., Jun, Z., Hongmei, G. and Fan, Q. (2019) CFLAR is a critical regulator of cerebral ischaemia-reperfusion injury through regulating inflammation and endoplasmic reticulum (ER) stress. *Biomed Pharmacother*, **117**, 109155.

38. Xiao, J., Moon, M., Yan, L., Nian, M., Zhang, Y., Liu, C., Lu, J., Guan, H., Chen, M., Jiang, D. *et al.* (2012) Cellular FLICE-inhibitory protein protects against cardiac remodelling after myocardial infarction. *Basic Res Cardiol*, **107**, 239.

39. Liu, X., Li, X. and Yu, S. (2024) CFLAR: A novel diagnostic and prognostic biomarker in soft tissue sarcoma, which positively modulates the immune response in the tumor microenvironment. *Oncol Lett*, **27**, 151.

40. Sheng, C., Yao, C., Wang, Z., Chen, H., Zhao, Y., Xu, D., Huang, H., Huang, W. and Chen, S. (2018) Cyclophilin J limits inflammation through the blockage of ubiquitin chain sensing. *Nat Commun*, **9**, 4381.
